# The impact of transvenous cardioverter-defibrillator implantation on quality of life, depression and optimism in dialysis patients: report on the secondary outcome of QOL in the randomized controlled ICD2 trial

**DOI:** 10.1007/s11136-020-02744-7

**Published:** 2021-02-19

**Authors:** Rohit J. Timal, Veronique de Gucht, Joris I. Rotmans, Liselotte C. R. Hensen, Maurits S. Buiten, Mihaly K. de Bie, Hein Putter, Martin J. Schalij, Ton J. Rabelink, J. Wouter Jukema

**Affiliations:** 1 Department of Cardiology, Leiden University Medical Center, PO Box 9600, 2300 RC Leiden, The Netherlands; 2 Department of Health and Medical Psychology, Leiden University, Leiden, The Netherlands; 3 Department of Internal Medicine, Leiden University Medical Center, Leiden, The Netherlands; 4 Department of Cardiology, Treant Zorggroep, Hoogeveen, The Netherlands; 5 Department of Biomedical Data Sciences, Leiden University Medical Center, Leiden, The Netherlands

**Keywords:** Self-report, Questionnaire, QOL, Depression, Optimism, Dialysis, ICD

## Abstract

**Rationale:**

The impact of prophylactic implantable cardioverter-defibrillator (ICD) implantation on the psychological well-being of patients on dialysis is unknown.

**Objective:**

We aimed to identify the effect of primary ICD implantation on quality of life (QoL), mood and dispositional optimism in patients undergoing dialysis.

**Methods and results:**

We performed a prespecified subanalysis of the randomized controlled ICD2 trial. In total, 177 patients on chronic dialysis, with an age of 55–81 years, and a left ventricular ejection fraction of ≥ 35%, were included in the per-protocol analysis. Eighty patients received an ICD for primary prevention, and 91 patients received standard care. The Short Form-36 (SF-36), Geriatric Depression Scale-15 (GDS-15), Revised Life Orientation Test (LOT-R) questionnaires were administered prior to ICD implantation (T0), and at 1-year follow-up (T1) to assess QoL, depression and optimism, respectively. The patients were predominantly male (76.0%), with a median age of 67 years. Hemodialysis was the predominant mode of dialysis (70.2%). The GDS-15 score difference (T1 − T0) was 0.5 (2.1) in the ICD group compared with 0.3 (2.2) in the control group (mean difference − 0.3; 95% CI − 1.1 to 0.6; *P* = 0.58). The LOT-R score difference was − 0.2 (4.1) in the ICD group compared with − 1.5 (4.0) in the control group (mean difference − 1.1 (0.8); 95% CI − 2.6 to 0.4; *P* = 0.17). The mean difference scores of all subscales of the SF-36 were not significantly different between randomization groups.

**Conclusions:**

In our population of patients on dialysis, ICD implantation did not affect QoL, mood or dispositional optimism significantly during 1-year follow-up.

**Clinical Trial Registration:**

Unique identifier: ISRCTN20479861. http://www.controlled-trials.com.

**Supplementary Information:**

The online version of this article (10.1007/s11136-020-02744-7) contains supplementary material, which is available to authorised users.

## Plain English summary

We found that in patients on dialysis, implantation of an implantable cardioverter-defibrillator (ICD), which is a device that can detect and treat life-threatening heart rhythm disturbances, did not affect quality of life, mood or dispositional optimism significantly in the first year after implantation.

This new data is important because it can help physicians to decide what the best, possibly life-saving, treatment is for the approximately 2 million patients suffering from end-stage kidney disease worldwide. Unfortunately, kidney failure requiring chronic dialysis treatment is associated with an increased risk of death and poses a major negative effect on a patient's physical and mental well-being. Preventive implantation of an ICD is not uncommon in these patients. However, the physical and psychological impact of preventive ICD implantation in these vulnerable patients is unknown. It was our goal to shed light on this by using three questionnaires (SF-36, GDS-15 and LOT-R) to map the physical and mental condition of 177 dialysis patients with an age of 55–81 years, with moderate to normal cardiac function. After filling out the questionnaires, 80 patients received an ICD, while the remaining 91 patients received standard care. One year later, all patients were asked again to complete the same three questionnaires. The results of the patients with an ICD were compared with the patients without an ICD, and we found no differences between both groups.

## Introduction

The incidence of end-stage renal disease (ESRD) in the United States in 2017 was 370 per million/year [1]. ESRD poses a high burden of disease, causing physical and psychological harm [2–5]. Dialysis treatment, although life-saving, is often perceived as burdensome and negatively impacts quality of life (QOL); patients on chronic dialysis are at high risk of anxiety and depression [2, 6–12]. Patients treated with dialysis are reported to be at high risk of malignant ventricular tachyarrhythmia, although reports in the literature likely overestimate sudden cardiac death incidence in this population [1, 13, 14]. Patients known to have life-threatening arrhythmias or sudden cardiac arrest survivors may be eligible for prophylactic use of an implantable cardioverter-defibrillator (ICD) to increase life expectancy [15–18]. ICD implantation can impact psychological well-being of ICD-patients by reduction of anxiety with regard to sudden cardiac death by providing reassurance or feelings of security. On the other hand, in a subgroup of ICD-recipients, an ICD is also known to cause adverse psychological events like anxiety and depression [19–22]. ICD-patients report that living with an ICD can introduce a number of physical and psychological adjustment issues, such as fear of defibrillation, fear of driving or (temporary) loss of driver’s license, fear of isolation, fear of device failure and fear of sexual- or physical activity [23]. Anxiety following device implantation could lead to alteration of leisure-time activities, e.g. refraining from enjoyed activities such as sports or hobbies, which in turn could lead to diminished QOL. Even partners of ICD patients can experience psychological distress levels as high as that of ICD-recipients [24]. An ICD shock can be experienced as extremely traumatizing. Likewise, dialysis treatment is known to cause psychological distress and to negatively impact QOL [25]. Comorbidity and the risk of complications in this vulnerable group make it unclear how these (potentially) life-saving therapies concomitantly affect physical and mental well-being, which are important but under-exposed variables. Our study group previously described the value of prophylactic ICD therapy in patients on chronic dialysis with left ventricular ejection fraction (LVEF) ≥ 35% [14]. Presently, however, the effect of an ICD implantation on the physical and mental state of patients on dialysis is unknown. The purpose of the current prespecified substudy of the randomized controlled ICD2 trial was to prospectively evaluate the impact of ICD implantation on QOL, mood or dispositional optimism in patients on maintenance dialysis.

## Methods

### Study design

In this report we prospectively evaluated the impact of ICD implantation on QOL, mood and dispositional optimism of patients participating in the Implantable Cardioverter-Defibrillator in Dialysis Patients (ICD2) trial [14]. Information regarding study design, randomization, study population, ICD implantation and device setup was described previously [14]. In short, ICD2 trial is an investigator-initiated, randomized, prospective, controlled study of ICD therapy versus no ICD in patients with LVEF ≥ 35% on chronic dialysis. Nephrologists from 17 dialysis centers in The Netherlands referred patients for screening to Leiden University Medical Center, The Netherlands. Following inclusion, patients were asked to fill in three well-established self-rating questionnaires to monitor changes in QOL, mood and dispositional optimism using the MOS 36-item short-form health survey (SF-36, version 1), Geriatric Depression Scale-15 (GDS-15) and the Revised Life Orientation Test (LOT-R), respectively. Completing the three mentioned questionnaires took approximately 30 min.

Patients allocated to the ICD group received a Biotronik (Berlin, Germany) transvenous device that was implanted subcutaneously, under local anesthesia, at Leiden University Medical Center, The Netherlands. Longitudinal assessment of physical and mental functioning, mood and optimism was performed after 1-year follow-up, using the same previously mentioned measurement tools (SF-36, GDS-15 and the LOT-R). Our study group previously demonstrated that prophylactic ICD therapy was not associated with a reduced rate of sudden cardiac death or all-cause death in dialysis patients. The findings of the ICD2 trial do not support routine ICD implantation to prevent death in dialysis patients with LVEF ≥ 35%. However, in case of a class I indication, such as a previous episode of ventricular fibrillation, device-implantation in these patients appeared feasible. The results of the ICD2 trial were not yet known during the conduct of the QOL substudy. The ICD2 trial protocol was approved by the ethics committee of Leiden University Medical Center in April 2007. The first patient was enrolled in June 2007 and the last in January 2018. The trial was stopped early (February 2018) on the advice of the data and safety monitoring board for futility reasons, after inclusion of 188 patients. The trial is registered at the Netherlands Trial Register (http://www.controlled-trials.com/ISRCTN20479861). All patients provided written informed consent.

### Population

Inclusion criteria were age ≥ 55 years and < 81 years, and ESRD treated with hemodialysis or peritoneal dialysis for ≥ 90 days. Patients on dialysis meeting the class I indication for ICD implantation were excluded. Also, patients with heart failure (New York Heart Association functional class IV) or a medical condition making 1-year survival unlikely were excluded [26, 27]. Patients with a central venous catheter were not eligible. Patients were also excluded if they were being prepared for a living kidney donation, had an acute myocardial infarction in the past 40 days, had human immunodeficiency virus, or were unable to give informed consent. Patients were randomized in a 1:1 fashion, to receive an ICD (ICD group) or usual care (control group).

Baseline data were collected concerning gender, age, BMI, blood pressure, smoking status, dialysis modality and dialysis vintage, cause of renal failure, and medication use. Additionally, information on cardiovascular comorbidity was documented.

### Questionnaires

#### MOS 36-item short-form health survey

Patients were asked to fill out the validated Dutch language version of the Medical Outcome Study Questionnaire Short Form 36 Health Survey (SF-36, version 1) to assess health-related QOL at baseline, and at 1 year follow-up (Supplement 1) [28, 29]. The SF-36 is a widely used measure of QOL and consists of eight subscales that are vitality, physical functioning, social functioning, bodily pain, general health perceptions, physical role functioning, emotional role functioning, and mental health. The SF-36 scores range from 0 to 100. Higher scores indicate better QOL.

#### Geriatric Depression Scale-15

Among the respondents depression was assessed using a Dutch version of the Geriatric Depression Scale-15 (GDS-15, Supplement 2) [30]. Patients were asked to answer this mood scale which consists of 15 questions in a *Yes/No* format. Items 1, 5, 7, 11 and 13 answered with *No* corresponded with depression while all other items scored a point for depression if answered with *Yes*. GDS-15 scores of 0–5 are in the normal range while a GDS scores of 6—10 are suggestive for mild depression and scores of 11 or greater are an indicator for major depression.

#### Revised Life Orientation Test

Optimism is a positive attitude about the future, otherwise described as one’s tendency to expect positive outcomes. Dispositional optimism positively affects health outcomes and health-related behaviour, whilst the antonym pessimism is associated with negative outcomes such as hopelessness and depressive symptoms. The Revised Life Orientation Test (LOT-R) is a reliable and valid easy-to-use self-report measure to assess dispositional optimism [31]. Patients were assessed with the Dutch version of the LOT-R, which is a short 10-item Likert scale questionnaire which quantifies optimism (Supplement 3) [32]. Higher scores on the LOT-R correspond with higher levels of Optimism. Six out of 10 items of this questionnaire were analyzed; the remaining 4 items are distraction items, also known as fillers, and were excluded from statistical analyses. LOT-R items 1, 4 and 10 were related to Optimism, whilst items 3, 7 and 9 contributed to Pessimism.

#### Health status: visual analogue scale

On the visual analogue scale (VAS, Supplement 4) respondents were asked to choose a point on the straight line that corresponds to their state of health in the past week, where 0 is the worst health condition imaginable and 100 correlates with perfect health.

### Statistical analysis

Statistical analyses were performed according to the per-protocol principle, using SPSS version 25.0 (IBM Corp, Armonk, New York, USA). Categorical data are presented as absolute numbers (%) and continuous data as mean (SD), or median and interquartile range in case of a non-normal distribution. Analysis of each of the SF-36 domains was based on a simple (Welch) independent samples t-test, comparing changes in QOL outcomes between the two treatment arms. To compare mean LOT-R score differences (normally distributed data) between ICD and control group at the 2 time points, e.g. baseline and 1-year follow-up, an independent samples *t* test was used. Using relative risk (RR) statistical differences were tested for occurrence of depression according to the GDS-15 questionnaire. Statistical significance was tested using the Chi-square test. Data regarding VAS was also analyzed using the independent samples *t*-test. We did not use imputation methods for missing data for the univariate analysis.

The internal consistency of the questionnaires was assessed using Cronbach’s alpha. A Cronbach’s alpha coefficient of > 0.7 was considered evidence for scale reliability. Because both renal transplantation and repeated hospitalization can have an impact on QOL and psychological distress, a secondary analysis hierarchical multiple regression analysis was conducted, with change (from baseline to follow-up) in depression (GDS-15), Optimism (LOT-R) and QOL (the eight domains of the SF-36) as outcomes. Within each of the regression models, treatment arm was entered in block 1. We have included the variables ‘kidney transplant within 1 year following inclusion’ and ‘number of hospitalizations within 1 year following inclusion’ in block 2 of the regression model. To control for baseline levels of the outcome variables, the baseline level of the outcome variable was entered in the third and final block of the regression model. A *p* value below 0.05 was considered statistically significant.

Finally, as sensitivity analysis, to protect against possible missingness at random, we performed a linear mixed model analysis for each of the SF-36 domains, LOT-R and GDS-15, with time (categorical) and treatment and their interaction as fixed effects and with random person effects.

## Results

### Descriptives and univariate analyses

**Fig. 1:**
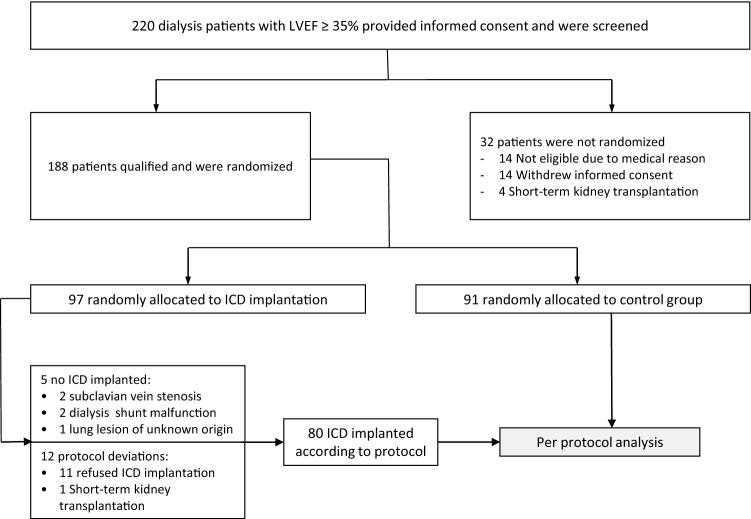
Study flow chart. ICD indicates implantable cardioverter defibrillator. *LVEF* left ventricular ejection fraction

Between July 2007 and January 2018, a total of 188 patients were enrolled in the ICD2 trial, of which 97 in the ICD group and 91 in the control group [14]. Patients that refused ICD implantation or patients who could not receive an ICD implantation after being randomized to the treatment group were excluded from the per-protocol analysis (*n* = 17). So, 171 patients were included in the current per-protocol analysis, of which 80 patients were allocated to the ICD group and 91 patients to the standard care group (Fig. 1). The baseline characteristics are depicted in Table 1. Males represented 76.0% of our population. The median age was 67 years (interquartile range [IQR] 62–74). Haemodialysis was the predominant mode of renal replacement therapy (70.2%). Table 1 also shows the baseline characteristics of the population with completed questionnaires at baseline and 1-year follow -up.

**Table 1:** Baseline characteristics

	Per-protocol population			Population with completed questionnaires at baseline and 1 year follow-up*		
	ICD Group (*n* = 80)	Control Group (*n* = 91)	*p* value	ICD group (*n* = 55)	Control Group (* n* = 58)	*p* value
Male, *n* (%)	61 (76.3)	69 (75.8)	0.95	41 (74.5)	47 (81.0)	0.41
Age, years, median (IQR)	67 (63–74)	68 (61–74)	0.68	67 (63–75)	67 (60–74)	0.90
Body mass index, kg/m^2^, mean (SD)	28.2 (5.6)	27.2 (4.7)	0.20	29.1 (5.5)	27.1 (4.4)	0.03
Heart rate, bpm, mean (SD)	70 (12)	73 (13)	0.14	68 (11)	73 (14)	0.03
Blood pressure, mmHg, mean (SD)						
Systolic	141 (23)	138 (21)	0.46	139 (21)	138 (23)	0.73
Diastolic	75 (11)	74 (11)	0.48	75 (11)	76 (10)	0.62
Dialysis						
Duration of dialysis, months, median (IQR)	16 (9–24)	15 (10–27)	0.95	16 (8–22)	15 (10–29)	0.91
Dialysis modality,* n* (%)			0.77			0.67
Haemodialysis	57 (71.3)	63 (69.2)		39 (70.9)	39 (67.2)	
Peritoneal dialysis	23 (28.8)	28 (30.8)		16 (29.1)	19 (32.8)	
*K*_t_/*V* urea/week			0.48			
Haemodialysis, mean (SD)	4.3 (3.6–4.9)	4.5 (3.8–5.1)		4.3 (1.0)	4.5 (0.9)	0.45
Peritoneal dialysis, mean (SD)	2.1 (1.9–2.5)	2.6 (2.1–3.4)		2.1 (0.5)	3.0 (0.7)	< 0.001
Symptoms,* n* (%)						
Angina pectoris	8 (10.0)	14 (15.4)	0.29	5 (9.1)	10 (17.2)	0.20
Palpitations	17 (21.3)	21 (23.1)	0.77	11 (20.0)	14 (24.1)	0.60
Oedema	7 (8.8)	12 (13.2)	0.36	4 (7.3)	8 (13.8)	0.26
Dyspnoea	30 (37.5)	42 (46.2)	0.25	20 (36.4)	28 (48.3)	0.20
Orthopnoea	7 (8.8)	5 (5.5)	0.41	5 (9.1)	3 (5.2)	0.48
Intermittent claudication	15 (18.8)	14 (15.4)	0.56	9 (16.4)	7 (12.1)	0.51
Medical history,* n* (%)						
Diabetes mellitus	27 (33.8)	38 (41.8)	0.28	20 (36.4)	21 (36.2)	0.99
Atrial fibrillation or flutter	20 (25.0)	17 (18.7)	0.32	15 (27.3)	12 (20.7)	0.41
Percutaneous transluminal coronary angioplasty	9 (11.3)	16 (17.6)	0.24	8 (14.5)	10 (17.2)	0.70
Coronary artery bypass graft	8 (10.0)	13 (14.3)	0.39	4 (7.3)	6 (10.3)	0.57
Myocardial infarction	16 (20.0)	27 (29.7)	0.15	12 (21.8)	17 (29.3)	0.36
Transient ischemic attack/cerebrovascular accident	13 (16.3)	18 (19.8)	0.55	6 (10.9)	9 (15.5)	0.47
Hypertension	66 (82.5)	71 (78.0)	0.46	45 (81.8)	47 (81.0)	0.92
Hypercholesterolemia	45 (56.3)	43 (47.3)	0.24	32 (58.2)	29 (50.0)	0.38
Cardiovascular risk profile,* n* (%)						
Family history of premature cardiovascular disease	28 (35.0)	30 (33.0)	0.78	18 (32.7)	21 (36.2)	0.70
Smoking,* n* (%)			0.39			0.09
Never	30 (37.4)	25 (27.5)		23 (41.8)	14 (24.1)	
Yes	17 (21.3)	26 (28.6)		10 (18.2)	18 (31.0)	
In the past	33 (41.3)	40 (44.0)		22 (40.0)	26 (44.8)	
Medication use,* n* (%)						
Beta-blocker	45 (56.3)	51 (56.0)	0.98	32 (58.2)	32 (55.2)	0.75
Angiotensin-converting enzyme inhibitor	15 (18.8)	19 (20.9)	0.73	12 (21.8)	10 (17.2)	0.54
Angiotensin receptor blocker	27 (33.8)	24 (26.4)	0.29	17 (30.9)	13 (22.4)	0.31
Calcium antagonist	30 (37.5)	29 (31.9)	0.44	20 (36.4)	17 (29.3)	0.43
Statin	47 (58.8)	58 (63.7)	0.50	35 (63.6)	38 (65.5)	0.83
Insulin	14 (17.5)	20 (22.0)	0.46	10 (18.2)	11 (19.0)	0.92
Erythropoietin	71 (88.8)	72 (79.1)	0.09	50 (90.9)	46 (79.3)	0.09
Cause of end-stage renal disease,* n* (%)			0.30			0.08
Diabetic nephropathy	20 (25.0)	21 (23.1)		14 (25.5)	9 (15.5)	
Hypertension	27 (33.8)	27 (29.7)		17 (30.9)	20 (34.5)	
Glomerulonephritis	13 (16.3)	9 (9.9)		11 (20.0)	5 (8.6)	
Other/unknown	20 (25.0)	34 (37.4)		13 (23.6)	24 (41.4)	
Echocardiography,* n* (%)						
Left ventricular ejection fraction (LVEF), %			0.14			0.13
LVEF ≥ 55%	51 (63.8)	45 (49.5)		39 (70.9)	32 (55.2)	
LVEF ≥ 45% and < 55%	21 (26.3)	30 (33.0)		13 (23.6)	17 (29.3)	
LVEF ≥ 35% and < 45%	8 (10.0)	16 (17.6)		3 (5.5)	9 (15.5)	
Left ventricular hypertrophy	37 (46.3)	43 (47.3)	0.90	28 (50.9)	25 (43.1)	0.41

*n* (%); *ICD* implantable cardioverter defibrillator; *K*_t_*/V* K dialyser clearance of urea, *t* dialysis time, *V* volume of distribution of urea, *LVEF* left ventricular ejection fraction

*Baseline characteristics of population in which prospective SF-36 questionnaire analysis was possible

During 1-year follow-up, 7.0% (12 out of 171) of the patients underwent kidney transplantation, 7 out of 80 (8.8%) in the ICD group and 5 out of 91 (5.5%) in the control group (relative risk 0.6; 95% CI 0.2–1.9; *p* = 0.41, Table 2). In the same period, mortality rates were 6.3% (5 out of 80) in the ICD group and 4.4% (4 out of 91) in the control group (relative risk 0.8; 95% CI 0.5–1.5; *p* = 0.59, Table 2). The most prevalent cause of death was of infectious origin. Among the 80 patients that underwent ICD implantation, 15 ICD-related adverse events occurred during 1-year follow-up. These adverse events were lead dysfunction/dislocation (*n* = 5), pocket infection (*n* = 4), pocket haematoma (*n* = 2), central venous stenosis (*n* = 2), lead perforation (*n* = 1) and inappropriate shock (*n* = 1). None of the patients in the ICD group received defibrillation therapy during 1-year follow-up.

**Table 2:** Clinical outcomes during 1-year follow-up

Clinical outcomes	ICD *n* = 80		Control *n* = 91		*P* value	Mean difference^®^ Relative risk^¥^	95% CI
	*n* (%)	Mean (SD)	*n* (%)	Mean (SD)			
Hospitalizations > 1 day	68	0.9 (1.2)	85	0.9 (1.2)	0.65*	0.1^®^	− 0.3 to 0.5
Day care	94	1.2 (1.5)	82	0.9 (1.3)	0.20*	− 0.3^®^	− 0.7 to 0.1
Shunt interventions	67	0.8 (1.5)	51	0.6 (1.1)	0.16*	− 0.3^®^	− 0.7 to 0.1
Hospitalizations total	162	2.0 (1.8)	167	1.8 (1.7)	0.48*	− 0.2^®^	− 0.7 to 0.3
Kidney transplantation	7 (8.8)	NA	5 (5.5)	NA	0.41**	0.6^¥^	0.2 to 1.9
ICD-related adverse events	15 (18.8)	NA	NA	NA	NA	NA	NA

*CI* confidence interval, *ICD* implantable cardioverter defibrillator, *N* number, *NA* not applicable, *SD* standard deviation

*Unpaired *t* test, **Chi^2^ test, ^®^Mean difference, ^¥^Relative risk

### Questionnaire analyses

The Cronbach’s coefficient alpha estimates for the questionnaires are presented in Table 3. The internal consistency reliability of all scales exceeded 0.70, with the exception of the LOT-R.

**Table 3:** Internal consistency reliability

Scale	Number of items	Cronbach’s alpha	
		Baseline	Follow-up
SF-36 Item			
1. Physical function	10	0.92	0.75
2. Role physical	4	0.88	0.91
3. Bodily pain	2	0.80	0.88
4. General health	5	0.74	0.72
5. Vitality	4	0.73	0.85
6. Social functioning	2	0.73	0.80
7. Role emotional	3	0.85	0.87
8. Mental health	5	0.78	0.72
9. Health change	1	Not applicable	Not applicable
Life Orientation Test-Revised			
Optimism	3^a^	0.50	0.53
Pessimism	3^b^	0.71	0.61
Geriatric Depression Scale-15			
	15	0.83	0.80

*LOT-R items 1, 4 and 10 are positively warded

^†^LOT-R items 3, 7 and 9 are negatively warded

**Fig. 2:**
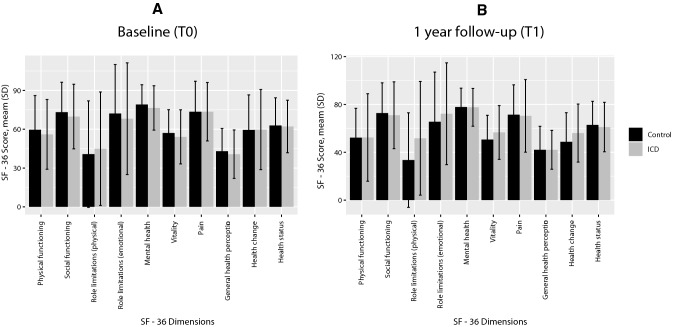
**a** SF-36, Baseline comparison of ICD group versus Control group. **b** SF-36, Follow-up comparison of ICD group versus Control group. T0 = mean (SD) scores at baseline, prior to potential ICD implantation; T1 = mean (SD) scores at 1-year follow-up

The response rates and results of the SF-36, GDS-15, VAS and LOT-R questionnaires are shown in Table 4. As expected, the mean scores of all SF-36 subcategories of patients randomized to receive an ICD and patients randomized for standard care, were similar at baseline (Fig. 2, Table 4). The changes in QOL outcomes regarding all SF-36 outcome variables, e.g. mean scores after a time interval of 1 year (T1) minus the baseline mean scores (T0), in the ICD group were statistically nonsignificant compared with changes in QOL outcomes in the control group (physical functioning (*p* = 0.43), social functioning (*p* = 0.89), physical role functioning (*p* = 0.49), emotional role functioning (*p* = 0.84), mental health (*p* = 0.91), vitality (*p* = 0.12), bodily pain (*p* = 0.61), general health perceptions (*p* = 0.85), health change (*p* = 0.50) and health status (*p* = 0.97)). The GDS-15 score difference (GDS-15 score at T1 minus GDS-15 score at T0) was 0.5 (2.1) in the ICD group compared with 0.3 (2.2) in the control group (mean difference − 0.3 (2.2); 95% CI -1.1 to 0.6; *p* = 0.58). The LOT-R score difference (LOT-R score at T1 minus LOT-R score at T0) was − 0.2 (4.1) in the ICD group compared with − 1.5 (4.0) in the control group (mean difference − 1.1 (0.8); 95% CI − 2.6 to 0.4; *p* = 0.17). The VAS score difference (VAS score at T1 minus VAS score at T0) was − 1.3 (19.7) in the ICD group compared with − 1.5 (21.8) in the control group (mean difference 0.2; 95% CI − 7.8 to 8.2; *p* = 0.97).

**Table 4:** Scores of the SF-36, health status, GDS-15 and the LOT-R

Questionnaire Scores	ICD group (*n* = 80)			Control group (*n* = 91)			Mean difference*	∆ 95% CI	*p*^†^
	Score at baseline (T0)	Scores at 12 months (*T*1)	∆T1 − T0	Score at baseline (T0)	Scores at 12 months (T1)	∆T1 − T0			
SF-36 scores, mean (SD)									
Response rate,* n* (%)	72 (90.0)	62 (77.5)	NA	82 (90.1)	61 (67.0)	NA	NA	NA	NA
Physical functioning	56.5 (27.3)	52.8 (38.0)	− 3.7 (29.8)	60.8 (26.4)	53.3 (25.4)	− 7.6 (20.2)	3.9	− 5.9 to 13.7	0.43
Social functioning	73.0 (22.5)	71.8 (28.1)	− 1.1 (27.0)	73.5 (21.3)	73.0 (26.2)	-0.4 (27.8)	− 0.7	− 11.0 to 9.6	0.89
Physical role limitations	47.0 (43.6)	52.5 (47.4)	5.5 (45.5)	34.7 (41.0)	34.7 (40.8)	0 (34.3)	5.5	− 10.3 to 21.3	0.49
Emotional role limitations	71.4 (42.5)	72.1 (42.1)	0.7 (48.3)	67.9 (40.8)	66.7 (40.8)	− 1.2 (46.7)	1.9	− 16.8 to 20.6	0.84
Mental health	79.2 (15.1)	77.5 (16.5)	− 1.7 (17.3)	79.9 (15.7)	78.5 (15.6)	− 1.4 (14.7)	− 0.3	− 5.7 to 6.4	0.91
Vitality	57.9 (19.4)	57.6 (23.2)	− 0.3 (20.6)	56.7 (17.5)	50.5 (20.7)	− 6.1 (18.4)	5.9	− 1.5 to 13.2	0.12
Bodily pain	75.1 (23.0)	70.6 (31.0)	− 4.4 (27.5)	74.3 (21.7)	72.3 (25.2)	− 2.0 (23.6)	− 2.5	− 12.1 to 7.2	0.61
General health perceptions	42.3 (17.0)	41.3 (16.4)	− 1.1 (15.0)	42.4 (18.2)	41.9 (19.6)	− 0.4 (18.5)	− 0.6	− 7.0 to 5.8	0.85
Health change	60.5 (29.1)	54.5 (24.6)	− 5.9 (33.0)	58.3 (27.7)	48.2 (24.0)	− 10.1 (32.3)	4.2	− 8.1 to 16.4	0.50
Health status (VAS), mean (SD)	62.6 (19.3)	61.3 (20.5)	− 1.3 (19.7)	65.1 (19.3)	63.6 (20.6)	− 1.5 (21.8)	0.2	− 7.8 to 8.2	0.97
Response rate,* n* (%)	70 (87.5)	61 (76.3)	NA	77 (84.6)	62 (68.1)	NA	NA	NA	NA
GDS-15 Score‡, mean (SD)	2.9 (2.8)	3.0 (2.7)	0.5 (2.1)	3.2 (3.1)	3.1 (2.9)	0.3 (2.2)	− 0.3	− 1.1 to 0.6	0.58
Response rate	63 (78.8)	61 (76.3)	NA	71 (78.0)	61 (67.0)	NA	NA	NA	NA
No depression,* n* (%)	54 (67.5)	54 (67.5)	NA	58 (63.7)	53 (58.2)	NA	NA	NA	NA
Mild depression,* n* (%)	7 (8.8)	5 (6.3)	NA	9 (9.9)	6 (6.6)	NA	NA	NA	NA
Major depression, (%)	2 (2.5)	2 (2.5)	NA	4 (4.5)	2 (2.2)	NA	NA	NA	NA
LOT-R score^§^, mean (SD)	14.6 (3.4)	14.6 (3.0)	− 0.2 (4.1)	14.8 (3.3)	13.6 (3.1)	− 1.5 (4.0)	− 1.1 (0.8)	− 2.6 to 0.4	0.17
Response rate,* n* (%)	61 (76.3)	61 (76.3)	NA	71 (78.0)	60 (65.9)	NA	NA	NA	NA

*T0* score at baseline (prior to potential ICD implantation), mean (SD), *T1*  score at 1-year follow-up, mean (SD), ∆*T1* − *T0* score difference, mean (SD); *∆ 95% CI* 95% confidence interval of score difference, *n* number, *NA* not applicable, *SD* standard deviation, *SF-36* Medical Outcome Study Questionnaire Short Form 36 Health Survey, *GDS-15* Geriatric Depression Scale-15, *LOT-R* Revised Life Orientation Test, *VAS* visual analogue scale

*Mean difference = ∆T1 − T0 ICD group minus ∆T1 − T0 control group

^†^Welch two sample *t* test of ∆T1 − T0 control group versus ∆T1 − T0 ICD group

^‡^No Depression was defined as a GDS score of 0 to 5. Mild Depression was defined as a GDS score of 6 to 10. Major Depression was defined as a GDS score of 10 to 15

^§^On the LOT-R scale from 0 to 24, patients with low scores are considered pessimists, whilst patients with high scores are deemed optimists. There are no cut-off points on the pessimism-optimism continuum

### Regression analyses

The results of the regression analyses are reported in Table 5. For Depression, the total variance explained by the final model (including all three blocks) was 3.5%. The only variable that significantly contributed to changes in depression from baseline to follow-up, was having a kidney transplantation during the 1-year follow-up period (p = 0.047), with patients who had a transplantation reporting less depression at follow-up. For Optimism, the total variance explained by the final model was 37.6%. The only significant predictor was baseline level of Optimism (*p* < 0.001). For the Vitality subscale of the SF-36, the total variance explained by the final model was 14%. Two variables significantly predicted changes in vitality from baseline to follow-up. The strongest predictor was Vitality at baseline (*p* < 0.001), followed by having a transplantation during the follow-up period, with patients who had a transplantation reporting higher levels of Vitality at follow-up (*p* = 0.02). For Physical Functioning, Social Functioning, Physical Role Functioning, Emotional Role Functioning, Mental Health, and Bodily Pain, the total variance explained by the final model was 6%, 14%, 18%, 30%, 22%, and 7.3%, respectively. For each of these outcomes, the baseline level of the outcome was the only significant predictor of changes from baseline to follow-up (*p* = 0.01, *p* = 0.001, *p* = 0.001, *p* < 0.001,* p* < 0.001, and *p* = 0.001, respectively). Finally, the final model explained 25.1% of the variance with respect to changes in perceived General Health from baseline to follow up. Both General Health Perception at baseline (*p* < 0.001) and receiving a kidney transplantation during the follow-up period (*p* = 0.001) significantly predicted the outcome, with patients receiving a transplantation reporting better General Health at 1-year follow-up.

**Table 5:** Hierarchical multiple regression analyses

	∆ Depression			∆ Optimism			∆ Vitality			∆ Phys Funct			∆ Social Funct		
	ICD *n* = 48		Control *n* = 47	ICD *n* = 47		Control *n* = 47	ICD *n* = 55		Control *n* = 57	ICD *n* = 54		Control *n* = 52	ICD *n* = 55		Control *n* = 57
	*R*^2^	*β*	SE	*R*^2^	*β*	SE	*R*^2^	*β*	SE	*R*^2^	*β*	SE	*R*^2^	*β*	SE
Block 1: treatment arms	0.00			0.02			0.02			0.00			0.00		
ICD versus control		0.06	0.44		0.12	0.69		0.13	3.48		0.05	4.87		− 0.02	4.83
Block 2: medical control variables	0.04			0.02			0.05			0.03			0.01		
Renal transplantation		− 0.21*	0.83		0.11	1.16		0.21*	6.75		0.09	9.18		0.04	9.39
Hospitalizations		0.00	0.14		− 0.16	0.19		− 0.00	1.02		− 0.17	1.42		− 0.03	1.44
Block 3	0.04			0.37			0.10			0.06			0.17		
Baseline of outcome		− 0.19	0.09		− 0.61***	0.09		− 0.32***	0.10		− 0.25**	0.09		− 0.42***	0.11
*R*^2^ (adj.)	0.08 (0.04)			0.40 (0.38)			0.17 (0.14)			0.10 (0.06)			0.17 (0.14)		

	∆ Role Lim Phys			∆ Role Lim Emo			∆ Mental Health			∆ Pain			∆ General Health		
	ICD *n* = 50		Control *n* = 54	ICD *n* = 49		Control *n* = 53	ICD *n* = 55		Control *n* = 57	ICD *n* = 55		Control *n* = 57	ICD *n* = 52		Control *n* = 57
	*R*^2^	*β*	SE	*R*^2^	*β*	SE	*R*^2^	*β*	SE	*R*^2^	*β*	SE	*R*^2^	*β*	SE
Block 1: treatment arms	0.01			0.00			0.00			0.00			0.00		
ICD versus control		0.15	7.27		0.04	7.92		− 0.01	2.70		− 0.05	4.69		− 0.06	2.83
Block 2: medical control variables	0.03			0.01			0.02			0.00			0.11		
Renal transplantation		− 0.06	13.56		0.03	14.80		− 0.02	5.25		0.03	9.11		0.28**	5.47
Hospitalizations		− 0.14	2.11		− 0.00	2.33		− 0.14	0.79		− 0.07	1.38		− 0.06	0.84
Block 3	0.18			0.32			0.23			0.10			0.17		
Baseline of outcome		− 0.43***	0.09		−0.58***	0.10		− 0.48***	0.09		− 0.32**	0.11		− 0.42***	0.08
*R*^2^ (adj.)	0.21 (0.18)			0.33 (0.30)			0.25 (0.22)			0.11 (0.07)			0.28 (0.25)		

∆ change score between baseline and follow-up

*β* final model, including all three blocks, *Vitality* Vitality subscale of the SF-36, *Phys Funct* Physical Functioning subscale of the SF-36, *Social Funct* Social functioning subscale of the SF-36, *Role Lim Phys* Role Limitations Physical subscale of the SF-36, *Role Lim Emo* Role Limitations Emotional, *Mental Health* Mental Health subscale of the SF-36, *Pain* Pain subscale of the SF-36, *General Health* General Health subscale of the SF-36

*p < 0.05; **p < 0.01; ***p < 0.001

### Sensitivity analysis

The main purpose of this sensitivity analysis was to check whether the results of differences in changes in QOL outcomes between the two treatment arms in the simple t-test coincided with that of the interaction in the linear mixed model. For all QOL outcomes the differences in changes in QOL outcomes between the treatment arms were both quantitatively (in terms of effect sizes) and quantitatively (in terms of statistical significance) similar (eSupplement 1, SPSS output).

## Discussion

Previously we reported on the quantitative data of the impact of ICD therapy in patients undergoing dialysis, with respect to survival and adverse effects, by executing a randomized clinical trial [14]. In this paper, we report on the longitudinal assessment of the physical and mental disease burden of ICD-recipients on dialysis (*n* = 80) versus patients undergoing dialysis without an ICD (*n* = 91), using three validated self-report questionnaires. In this per-protocol subanalysis of the ICD2 trial we found that in ESRD patients undergoing dialysis, ICD implantation did not (negatively or positively) affect QOL, mood or dispositional optimism in the first year post-implantation. Multiple regression analysis showed that a kidney transplantation during the 1-year follow-up period significantly contributed to changes in depression from baseline to follow-up (p = 0.047), with patients who had a transplantation reporting less depression at follow-up. Additionally, patients receiving a transplantation reported better General Health at 1-year follow-up.

To the best of our knowledge, we are the first to report on the impact of an ICD on QOL in this population. Early recognition (of patients at high risk) of adverse psychological events is of paramount importance for timely intervention. In comparison with other reports of QOL in ICD-recipients or reports of QOL among patients on dialysis, our trial consists of a relatively large population (*n* = 188) [21, 22]. Also, the follow-up duration of the current study of 12 months offers valuable insights. Moreover, our trial design included a proper control group. Also, we assessed the preimplantation scores of the three questionnaires, which is an important strength of this trial. Reliable and valid screening devices were used for measuring mental well-being and certain traits that influence coping behaviour. The fact that we have not been able to demonstrate an effect of ICD implantation on the mentioned scales does not seem to be based on an underpowerment of this study. As we are the first to perform a prospective evaluation of the effect of ICD therapy on the physical and mental state of patients with ESRD we cannot compare our results with other reports from the literature.

Multiple studies evaluating QOL in patients on dialysis report high rates of depression and anxiety [6, 7, 33]. Kim et al. assessed QOL among hemodialysis patient and found similar health-related QOL scores compared to our results [33]. Also, Nagasawa and colleagues evaluated the effect of QOL on medication compliance among 92 dialysis patients and found QOL-scores comparable to QOL-scores in this report [7]. Adaptation distress among ICD-recipients in the general population has also been described in several reports [11, 23]. However, these reports mostly concern cross-sectional data, or longitudinal data with small sample size, and are lacking a proper control group without an ICD. Also, consensus regarding the relationship of QOL and ICD implantation is lacking because of equivocal trial outcomes [11, 21–23, 34]. Kamphuis et al. assessed QOL and depressive symptoms in a 12-month longitudinal study among 132 ICD-recipients versus 35 patients without ICD and found clinically significant depressive symptoms in 22–66% of ICD recipients throughout the first year [21]. Lewin et al. conducted an RCT to assess the effect of cognitive behavioural rehabilitation on QOL and depression in 192 patients scheduled to receive an ICD and after 6 months following ICD-implantation [35]. However, this study group did not include a control group that did not undergo ICD-implantation. Also, both randomization groups were not comparable with regard to baseline QOL and depression scores [35]. Friedman et al. mapped the QOL and anxiety among 48 ICD recipients in a cross-sectional study and found that younger patients were at the highest risk of psychological distress and poor QOL [22]. Irvine et al. performed a randomized controlled trial to assess the impact of ICD implantation on QOL among 317 patients known with sustained ventricular arrhythmias randomized to receive ICD implantation (*n* = 157) or amiodarone (*n* = 160). Authors found, after a follow-up of 12 months, that QOL improved in the ICD group [36]. Conversely, Schron et al. compared QOL outcomes among 416 patients randomized for ICD implantation versus 384 patients receiving anti-arrhythmic drugs and found no significant difference over 1-year follow-up [37]. Magyar-Russel et al. reviewed 42 studies that assessed (symptoms of) depression among ICD recipients, of which only 5 were longitudinal cohort reports, all suggesting no change over time [11]. Fitchet et al. performed an RCT among 12 ICD recipients and found 12 week cardiac rehabilitation can improve anxiety and depression rates [38]. Sears et al. performed an RCT to assess the effect of psychological treatment on depression and QOL among 30 ICD-recipients that received an ICD-shock within 1 year following device implantation. The investigators found that depression, and QOL improved significantly from baseline to postintervention in all participants and concluded that structured interventions for shocked ICD patients involving ICD education and cognitive-behavioural strategies can reduce psychological distress and improve QOL [20].

Our trial had some limitations. Firstly, as in any trial, we had patients from the main trial that did not want to participate in this substudy. The baseline characteristics of the subgroup with completed questionnaires at baseline and 1-year follow-up are depicted in Table 1, in addition to the baseline characteristics of the complete per protocol population. This table shows that both populations are very similar, as expected. Secondly, although the LOT-R is a widely used, and well-validated questionnaire to measure dispositional Optimism, we found a low internal validity in our population, especially for the subscale measuring Optimism. As can be seen in Table 3 the Cronbach’s alphas for Optimism (*α* = 0.50 at baseline, *α* = 0.53 at follow up) and Pessimism at follow up (*α* = 0.61) yielded a lower internal consistency than expected. One explanation for this is the limited number of items the subscales consist of. An additional explanation for the low internal consistency of the subscale Optimism, both at baseline and at follow-up, is the fact that the intercorrelations between the items the subscale consists of are quite low. Thirdly, absence of significant findings may be the result of the limited sample size. However, with respect to reports in literature, our population is relatively large and has a control group. Also, we performed a sensitivity analysis and found that for all QOL outcomes the differences in changes in QOL outcomes between the treatment arms were both quantitatively (in terms of effect sizes) and quantitatively (in terms of statistical significance) similar. Furthermore, our population consisted of a highly selective patient group. Consequently, results may not be generalizable to the overall dialysis/ICD population. However, we found approximately equal levels of QOL in our dialysis population compared with reports in the literature [5, 24, 33]. Also, we did not include patients with a class I indication for ICD implantation according to the current guidelines. The long-term effect of ICD implantation in dialysis patients has not been charted, as follow up in our trial was performed following a time span of 1 year. The poor prognosis of this vulnerable patient group would lead to high rate of missing values because of high mortality in case of longer follow-up. Furthermore, prospective collection of multiple extensive questionnaires can be experienced as bothersome by patients, which does not benefit the response rate. However, distress levels may change over time. It could be hypothesized that after longer follow up an ICD could provide feelings of safety and results in a better QOL due to less anxiety through better adaptation. Conversely, feelings of anxiety for imminent shock and occurrence of adverse events, could lead to a further decrease in QOL after longer follow-up. We did not map the social support of patients.

In conclusion, in the current trial we shed light on the physical and psychological well-being of ESRD patients on dialysis undergoing prophylactic ICD implantation for primary prevention. Our data suggests that ICD implantation does not seem to influence QOL, negatively or positively, among dialysis patients during 1-year follow-up. Based on these results, we believe that our study provides evidence that in case of a hard indication for preventive ICD implantation, this procedure should not be withheld in this population. Further study is warranted for evaluation of long-term implication of an ICD in this vulnerable patient group.

## Supplementary Information

Below is the link to the electronic supplementary material.

Supplementary file 1 (JPEG 5262 kb)

Supplementary file 2 (DOCX 163 kb)

Supplementary material 3 (PDF 717 KB)

## Data Availability

The data that support the findings of this study are available from the corresponding author upon reasonable request. Code availability The SPSS syntaxes used for the analysis of this study are available from the corresponding author upon reasonable request.
