# Supplementary figures and images for: The impact of transvenous cardioverter-defibrillator implantation on quality of life, depression and optimism in dialysis patients: report on the secondary outcome of QOL in the randomized controlled ICD2 trial
Source: Qual Life Res. 2021 Feb 19;30(6):1605–17. doi: 10.1007/s11136-020-02744-7 (PMC8178151; doi:10.1007/s11136-020-02744-7)

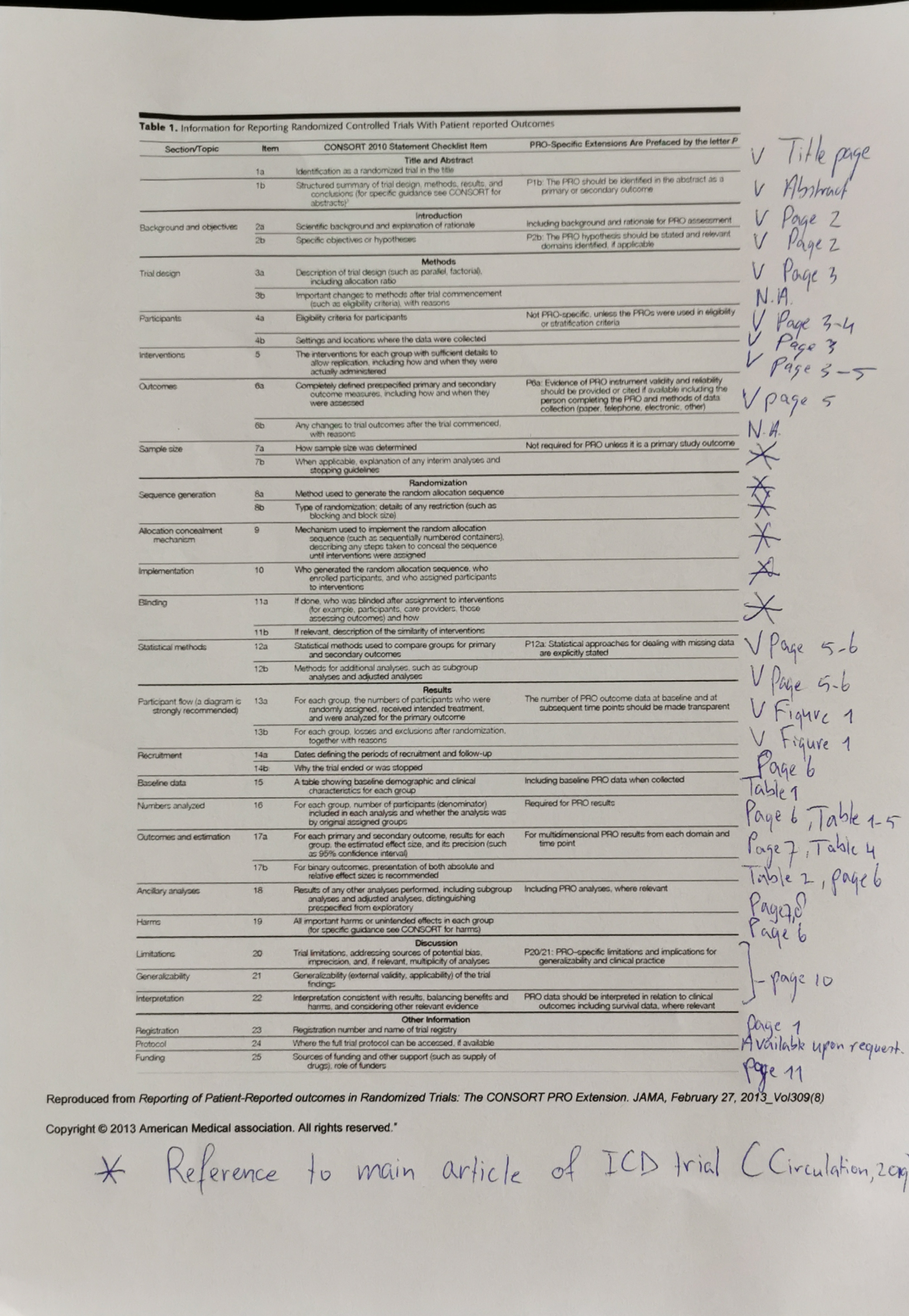

Supplement: Supplementary file 1 — (JPEG 5262 kb) [file 11136_2020_2744_MOESM1_ESM.jpg]
