# Supplementary material for: The impact of transvenous cardioverter-defibrillator implantation on quality of life, depression and optimism in dialysis patients: report on the secondary outcome of QOL in the randomized controlled ICD2 trial
Source: Qual Life Res. 2021 Feb 19;30(6):1605–17. doi: 10.1007/s11136-020-02744-7 (PMC8178151; doi:10.1007/s11136-020-02744-7)
Supplement: Supplementary file 2 — (DOCX 163 kb) [file 11136_2020_2744_MOESM2_ESM.docx]

**Supplement**

# The impact of transvenous cardioverter-defibrillator implantation on quality of life, depression and optimism in dialysis patients:

# Report on the secondary outcome of QOL in the randomized controlled ICD2 trial

**Rohit J. Timal^1^, MD; Veronique de Gucht^2^, PhD, Joris I. Rotmans^3^, MD, PhD; Liselotte C. R. Hensen^1^, MD; Maurits S. Buiten^1^, MD, PhD; Mihaly K. de Bie^1,4^, MD, PhD; Hein Putter^5^, PhD; Martin J. Schalij^1^, MD, PhD; Ton J. Rabelink^3^, MD, PhD; J. Wouter Jukema^1^, MD, PhD; for the ICD2 Trial Investigators†**

1. Department of Cardiology, Leiden University Medical Center, Leiden, The Netherlands.
2. Department of Health and Medical Psychology, Leiden University, Leiden, The Netherlands.
3. Department of Internal Medicine, Leiden University Medical Center, Leiden, The Netherlands.
4. Department of Cardiology, Treant Zorggroep, Hoogeveen, The Netherlands.
5. Department of Biomedical Data Sciences, Leiden University Medical Center, Leiden, The Netherlands.

**Keywords**: self-report, questionnaire, QOL, depression, optimism, dialysis, ICD.

**CLINICAL TRIAL REGISTRATION:** URL: <http://www.controlled-trials.com>.

Unique identifier: ISRCTN20479861.

**Correspondence**

Rohit J. Timal, MD, Department of Cardiology, Leiden University Medical Center, PO Box 9600, 2300 RC Leiden, The Netherlands. Tel.: +31715262020; Fax: +31715266809; Email: [rj_timal@hotmail.com](mailto:rj_timal@hotmail.com). https://orcid.org/0000-0003-0343-8043

Table of Contents

[Supplement 3](#_Toc40132372)

[Supplemental Methods 3](#_Toc40132373)

[Supplement 1. Medical Outcomes Study Questionnaire Short Form 36 Health Survey (SF-36), version 1 3](#_Toc40132374)

[Supplement 2. Geriatric Depression Scale (Short Form) 7](#_Toc40132375)

[Supplement 3. Revised Life Orientation Test (LOT-R) 8](#_Toc40132376)

[Supplement 4. Health Status: Visual Analog Scale 8](#_Toc40132377)

[Author Information 9](#_Toc40132378)

# Supplement

## Supplemental Methods

### Supplement 1. Medical Outcomes Study Questionnaire Short Form 36 Health Survey (SF-36), version 1

This survey asks for your views about your health. This information will help keep track of how you feel and how well you are able to do your usual activities. Thank you for completing this survey! For each of the following questions, please circle the number that best describes your answer.

1. **In general, would you say your health is:**

| Excellent | 1 |
| --- | --- |
| Very good | 2 |
| Good | 3 |
| Fair | 4 |
| Poor | 5 |

**2. Compared to one year ago,**

| Much better now than one year ago | 1 |
| --- | --- |
| Somewhat better now than one year ago | 2 |
| About the same | 3 |
| Somewhat worse now than one year ago | 4 |
| Much worse now than one year ago | 5 |

1. **The following items are about activities you might do during a typical day. Does your health now limit you in these activities? If so, how much?**

|  |  | **Yes, Limited a**  **Lot (1)** | **Yes, Limited a**  **Little (2)** | **No, Not limited at**  **All (3)** |
| --- | --- | --- | --- | --- |
| a | Vigorous activities, such as running, lifting  heavy objects, participating in strenuous sports | 1 | 2 | 3 |
| b | Moderate activities, such as moving a table,  pushing a vacuum cleaner, bowling, or playing golf | 1 | 2 | 3 |
| c | Lifting or carrying groceries | 1 | 2 | 3 |
| d | Climbing several flights of stairs | 1 | 2 | 3 |
| e | Climbing one flight of stairs | 1 | 2 | 3 |
| f | Bending, kneeling, or stooping | 1 | 2 | 3 |
| g | Walking more than a mile | 1 | 2 | 3 |
| h | Walking several blocks | 1 | 2 | 3 |
| i | Walking one block | 1 | 2 | 3 |
| j | Bathing or dressing yourself | 1 | 2 | 3 |

1. **During the past 4 weeks, have you had any of the following problems with your work or other regular daily activities as a result of your physical health?**

|  |  | **Yes (1)** | **No (2)** |
| --- | --- | --- | --- |
| a | Cut down the amount of time you spent on work or other activities | 1 | 2 |
| b | Accomplished less than you would like | 1 | 2 |
| c | Were limited in the kind of work or other activities | 1 | 2 |
| d | Had difficulty performing the work or other activities (for example, it took extra effort) | 1 | 2 |

1. **During the past 4 weeks, have you had any of the following problems with your work or other regular daily activities as a result of any emotional problems (such as feeling depressed or anxious)?**

|  |  | **Yes** | **No** |
| --- | --- | --- | --- |
| a | Cut down the amount of time you spent on work or other activities | 1 | 2 |
| b | Accomplished less than you would like | 1 | 2 |
| c | Didn't do work or other activities as carefully as usual | 1 | 2 |

**6. During the past 4 weeks, to what extent has your physical health or emotional problems interfered with your normal social activities with family, friends, neighbours, or groups?**

| Not at all | 1 |
| --- | --- |
| Slightly | 2 |
| Moderately | 3 |
| Quite a bit | 4 |
| Extremely | 5 |

1. **How much bodily pain have you had during the past 4 weeks?**

| None | 1 |
| --- | --- |
| Very mild | 2 |
| Mild | 3 |
| Moderate | 4 |
| Severe | 5 |
| Very severe | 6 |

1. **During the past 4 weeks, how much did pain interfere with your normal work (including both work outside the home and housework)?**

| Not at all | 1 |
| --- | --- |
| A little bit | 2 |
| Moderately | 3 |
| Quite a bit | 4 |
| Extremely | 5 |

These questions are about how you feel and how things have been with you **during the past 4 weeks**. For each question, please give the one answer that comes closest to the way you have been feeling.

1. **How much of the time during the past 4 weeks . . .**

|  |  | **All of the Time** | **Most of the Time** | **A Good Bit of the Time** | **Some of the Time** | **A Little of the Time** | **None of the Time** |
| --- | --- | --- | --- | --- | --- | --- | --- |
| a | Did you feel full of pep? | 1 | 2 | 3 | 4 | 5 | 6 |
| b | Have you been a very nervous person? | 1 | 2 | 3 | 4 | 5 | 6 |
| c | Have you felt so down in the dumps that nothing could cheer you up? | 1 | 2 | 3 | 4 | 5 | 6 |
| d | Have you felt calm and peaceful? | 1 | 2 | 3 | 4 | 5 | 6 |
| e | Did you have a lot of energy? | 1 | 2 | 3 | 4 | 5 | 6 |
| f | Have you felt downhearted and blue? | 1 | 2 | 3 | 4 | 5 | 6 |
| g | Did you feel worn out? | 1 | 2 | 3 | 4 | 5 | 6 |
| h | Have you been a happy person? | 1 | 2 | 3 | 4 | 5 | 6 |
| i | Did you feel tired? | 1 | 2 | 3 | 4 | 5 | 6 |

**10. During the past 4 weeks, how much of the time has your physical health or emotional problems interfered with your social activities (like visiting with friends, relatives, etc.)?**

| All of the time | 1 |
| --- | --- |
| Most of the time | 2 |
| Some of the time | 3 |
| A little of the time | 4 |
| None of the time | 5 |

**11. How TRUE or FALSE is each of the following statements for you.**

|  |  | **Definitely True** | **Mostly True** | **Don't Know** | **Mostly False** | **Definitely False** |
| --- | --- | --- | --- | --- | --- | --- |
| a | I seem to get sick a little easier than other people | 1 | 2 | 3 | 4 | 5 |
| b | I am as healthy as anybody I know | 1 | 2 | 3 | 4 | 5 |
| c | I expect my health to get worse | 1 | 2 | 3 | 4 | 5 |
| d | My health is excellent | 1 | 2 | 3 | 4 | 5 |

### Supplement 2. Geriatric Depression Scale (Short Form)


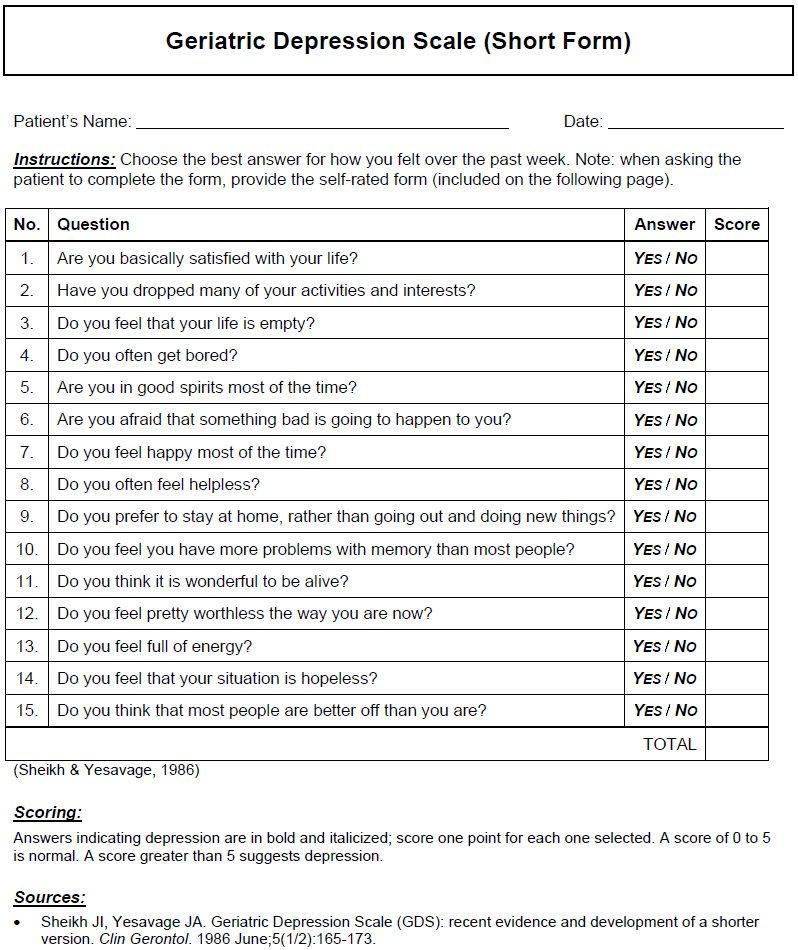


### Supplement 3. Revised Life Orientation Test (LOT-R)

***Instructions***: Please respond as accurately and honestly as you can. There are no right or wrong answers. Please circle your answer below.

|  |  | 1 | 2 | 3 | 4 | 5 |
| --- | --- | --- | --- | --- | --- | --- |
|  |  | Strongly disagree | Disagree | Neutral | Agree | Strongly agree |
| 1 | In uncertain times, I usually expect the best |  |  |  |  |  |
| 2 | It’s easy for me to relax. |  |  |  |  |  |
| 3 | If something can go wrong for me, it will |  |  |  |  |  |
| 4 | I’m always optimistic about my future |  |  |  |  |  |
| 5 | I enjoy my friends a lot |  |  |  |  |  |
| 6 | It’s important for me to keep busy |  |  |  |  |  |
| 7 | I hardly ever expect things to go my way |  |  |  |  |  |
| 8 | I don’t get upset too easily |  |  |  |  |  |
| 9 | I rarely count on good things happening to me |  |  |  |  |  |
| 10 | Overall, I expect more good things to happen to me than bad. |  |  |  |  |  |

0 = strongly disagree; 1 = disagree; 2 = neutral; 3 = agree; 4 = strongly agree

### Supplement 4. Health Status: Visual Analog Scale

Health represents a total of physical, mental and social well-being. We would like to ask you to give an appreciation of your health. Please use the line below to indicate how good or bad you thought your overall health condition was in the past week. You can do that by making a cross (X) somewhere between 0 and 100, where 0 stands for dead and 100 for perfect health.

**100**

**Perfect Health**

**0**

**Death**

## Author Information

**Rohit J. Timal, MD;** Department of Cardiology, Leiden University Medical Center, Leiden, The Netherlands. [r.j.timal@lumc.nl](mailto:r.j.timal@lumc.nl)

**Veronique de Gucht, PhD;** Department of Health and Medical Psychology, Leiden University, Leiden, The Netherlands. [DEGUCHT@FSW.leidenuniv.nl](mailto:DEGUCHT@FSW.leidenuniv.nl)

**Joris I. Rotmans, MD, PhD;** Department of Internal Medicine, Leiden University Medical Center, Leiden, The Netherlands. [J.I.Rotmans@lumc.nl](mailto:J.I.Rotmans@lumc.nl)

**Liselotte C. R. Hensen, MD;** Department of Cardiology, Leiden University Medical Center, Leiden, The Netherlands. [L.C.R.Hensen@lumc.nl](mailto:L.C.R.Hensen@lumc.nl)

**Maurits S. Buiten, MD, PhD;** Department of Cardiology, Leiden University Medical Center, Leiden, The Netherlands. [M.S.Buiten@lumc.nl](mailto:M.S.Buiten@lumc.nl)

**Mihaly K. de Bie, MD, PhD;** Department of Cardiology, Leiden University Medical Center, Leiden, The Netherlands, Department of Cardiology, Treant Zorggroep, Hoogeveen, The Netherlands. [m.debie@treant.nl](mailto:m.debie@treant.nl)

**Hein Putter, PhD;** Department of Biomedical Data Sciences, Leiden University Medical Center, Leiden, The Netherlands. [H.Putter@lumc.nl](mailto:H.Putter@lumc.nl)

**Martin J. Schalij, MD, PhD;** Department of Cardiology, Leiden University Medical Center, Leiden, The Netherlands. [M.J.Schalij@lumc.nl](mailto:M.J.Schalij@lumc.nl)

**Ton J. Rabelink, MD, PhD;** Department of Internal Medicine, Leiden University Medical Center, Leiden, The Netherlands. [A.J.Rabelink@lumc.nl](mailto:A.J.Rabelink@lumc.nl)

**J. Wouter Jukema, MD, PhD;** Department of Cardiology, Leiden University Medical Center, Leiden, The Netherlands. [J.W.Jukema@lumc.nl](mailto:J.W.Jukema@lumc.nl)
