# Supplementary material for: The impact of transvenous cardioverter-defibrillator implantation on quality of life, depression and optimism in dialysis patients: report on the secondary outcome of QOL in the randomized controlled ICD2 trial
Source: Qual Life Res. 2021 Feb 19;30(6):1605–17. doi: 10.1007/s11136-020-02744-7 (PMC8178151; doi:10.1007/s11136-020-02744-7)
Supplement: Supplementary file 3 — Supplementary material 3 (PDF 717 KB) [file 11136_2020_2744_MOESM3_ESM.pdf]

```
* Encoding: UTF-8.
TEMPORARY.
SELECT IF (ICDcontrol<=2 AND exclusie.x="Nee").
MIXED rphysf BY time ICDcontrol
  /CRITERIA=CIN(95) MXITER(100) MXSTEP(10) SCORING(1) SINGULAR(0.000000000001) HCONVERGE(0,
    ABSOLUTE) LCONVERGE(0, ABSOLUTE) PCONVERGE(0.000001, ABSOLUTE)
  /FIXED=time ICDcontrol time*ICDcontrol | SSTYPE(3)
  /METHOD=REML
  /PRINT=DESCRIPTIVES G  SOLUTION
  /RANDOM=INTERCEPT | COVTYPE(VC)
  /EMMEANS=TABLES(time*ICDcontrol) .
```

Mixed Model Analysis

Notes

Output Created		23-AUG-2020 12:09:46
Comments		
Input	Data	M:\HP\Consult\HARTZ\Timal\ICD2\QOLlong.sav
	Active Dataset	DataSet3
	Filter	<none>
	Weight	<none>
	Split File	<none>
	N of Rows in Working Data File	342
Missing Value Handling	Definition of Missing	User-defined missing values are treated as missing.
	Cases Used	Statistics are based on all cases with valid data for all variables in the model.

Syntax		MIXED rphysf BY time ICDcontrol<br>/CRITERIA=CIN(95) MXITER(100) MXSTEP(10) SCORING(1) SINGULAR(0.000000000001) HCONVERGE(0, ABSOLUTE) LCONVERGE(0, ABSOLUTE) PCONVERGE(0.000001, ABSOLUTE)<br>/FIXED=time ICDcontrol time*ICDcontrol | SSTYPE(3)<br>/METHOD=REML<br>/PRINT=DESCRIPTIVES G SOLUTION<br>/RANDOM=INTERCEPT | COVTYPE(VC)<br>/EMMEANS=TABLES(time*ICDcontrol) .
Resources	Processor Time	00:00:00,03
	Elapsed Time	00:00:00,05

Descriptive Statistics

rphysf

time	ICDcontrol	Count	Mean	Standard Deviation	Coefficient of Variation
1	Control	80	59,6250	26,44286	44,3%
	ICD	72	56,0417	26,94462	48,1%
	Total	152	57,9276	26,65355	46,0%
2	Control	58	52,2414	24,55164	47,0%
	ICD	61	52,3770	36,59015	69,9%
	Total	119	52,3109	31,17603	59,6%
Total	Control	138	56,5217	25,83361	45,7%
	ICD	133	54,3609	31,66116	58,2%
	Total	271	55,4613	28,80762	51,9%

Model Dimension[a]

		Number of Levels	Covariance Structure	Number of Parameters
Fixed Effects	Intercept	1		1
	time	2		1
	ICDcontrol	2		1
	time * ICDcontrol	4		1
Random Effects	Intercept[b]	1	Variance Components	1
Residual				1
Total		10		6

a. Dependent Variable: rphysf.

b. As of version 11.5, the syntax rules for the RANDOM subcommand have changed. Your command syntax may yield results that differ from those produced by prior versions. If you are using version 11 syntax, please consult the current syntax reference guide for more information.

Information Criteria[a]

-2 Restricted Log Likelihood	2569,001
Akaike's Information Criterion (AIC)	2573,001
Hurvich and Tsai's Criterion (AICC)	2573,046
Bozdogan's Criterion (CAIC)	2582,175
Schwarz's Bayesian Criterion (BIC)	2580,175

The information criteria are displayed in smaller-is-better form.

a. Dependent Variable: rphysf.

Fixed Effects

Type III Tests of Fixed Effects[a]

Source	Numerator df	Denominator df	F	Sig.

Intercept	1	,000	5,153	,000
time	1	267	2,452	,119
ICDcontrol	1	267	,239	,626
time * ICDcontrol	1	267	,278	,599

a. Dependent Variable: rphysf.

Estimates of Fixed Effects[a]

Parameter	Estimate	Std. Error	df	t	Sig.	95% Confidence Interval Lower Bound	95% Confidence Interval Upper Bound
Intercept	52,377049	24,474707	,000	2,140	,000	3,351045	101,403053
[time=1]	3,664617	5,011883	267	,731	,465	-6,203223	13,532458
[time=2]	0[b]	0	.	.	.	.	.
[ICDcontrol=1]	-,135670	5,282030	267	-,026	,980	-10,535399	10,264059
[ICDcontrol=2]	0[b]	0	.	.	.	.	.
[time=1] * [ICDcontrol=1]	3,719003	7,056149	267	,527	,599	-10,173768	17,611774
[time=1] * [ICDcontrol=2]	0[b]	0	.	.	.	.	.
[time=2] * [ICDcontrol=1]	0[b]	0	.	.	.	.	.
[time=2] * [ICDcontrol=2]	0[b]	0	.	.	.	.	.

a. Dependent Variable: rphysf.

b. This parameter is set to zero because it is redundant.

Covariance Parameters

Estimates of Covariance Parameters[a]

Parameter		Estimate	Std. Error
Residual		829,492785	71,791327
Intercept	Variance	585,413059	68719476735,999990

a. Dependent Variable: rphysf.

Random Effect Covariance Structure (G)[a]

	Intercept
Intercept	585,413059

Variance Components
a. Dependent Variable: rphysf.

Estimated Marginal Means

time * ICDcontrol[a]

time	ICDcontrol	Mean	Std. Error	df	95% Confidence Interval Lower Bound	95% Confidence Interval Upper Bound
1	Control	59,625	24,409	,000	-1,686	120,936
	ICD	56,042	24,432	,000	-,495	112,578
2	Control	52,241	24,489	,000	5,477	99,006
	ICD	52,377	24,475	,000	3,351	101,403

a. Dependent Variable: rphysf.

```
TEMPORARY.
SELECT IF (ICDcontrol<=2 AND exclusie.x="Nee").
MIXED  rsocf BY time ICDcontrol
  /CRITERIA=CIN(95) MXITER(100) MXSTEP(10) SCORING(1) SINGULAR(0.000000000001) HCONVERGE(0,
    ABSOLUTE) LCONVERGE(0, ABSOLUTE) PCONVERGE(0.000001, ABSOLUTE)
  /FIXED=time ICDcontrol time*ICDcontrol | SSTYPE(3)
  /METHOD=REML
  /PRINT=DESCRIPTIVES G  SOLUTION
  /RANDOM=INTERCEPT | COVTYPE(VC)
  /EMMEANS=TABLES(time*ICDcontrol) .
```

Mixed Model Analysis

Notes

Output Created		23-AUG-2020 12:09:46
Comments		
Input	Data	M:\HP\Consult\HARTZ\Timal\ICD2\QOLlong.sav
	Active Dataset	DataSet3
	Filter	<none>
	Weight	<none>
	Split File	<none>
	N of Rows in Working Data File	342
Missing Value Handling	Definition of Missing	User-defined missing values are treated as missing.
	Cases Used	Statistics are based on all cases with valid data for all variables in the model.
Syntax		MIXED rsocf BY time ICDcontrol<br>/CRITERIA=CIN(95) MXITER(100) MXSTEP(10) SCORING(1) SINGULAR(0.000000000001) HCONVERGE(0,<br>ABSOLUTE) LCONVERGE(0, ABSOLUTE) PCONVERGE(0.000001, ABSOLUTE)<br>/FIXED=time ICDcontrol time*ICDcontrol | SSTYPE(3)<br>/METHOD=REML<br>/PRINT=DESCRIPTIVES G SOLUTION<br>/RANDOM=INTERCEPT | COVTYPE(VC)<br><br>/EMMEANS=TABLES(time*ICDcontrol) .
Resources	Processor Time	00:00:00,06
	Elapsed Time	00:00:00,09

Descriptive Statistics

rsocf

time	ICDcontrol	Count	Mean	Standard Deviation	Coefficient of Variation
1	Control	82	73,1707	23,16756	31,7%
	ICD	72	69,7917	24,97798	35,8%
	Total	154	71,5909	24,01119	33,5%
2	Control	61	72,7459	25,36280	34,9%
	ICD	62	70,9677	27,97597	39,4%
	Total	123	71,8496	26,61743	37,0%
Total	Control	143	72,9895	24,04196	32,9%
	ICD	134	70,3358	26,31288	37,4%
	Total	277	71,7058	25,15533	35,1%

Model Dimension[a]

		Number of Levels	Covariance Structure	Number of Parameters
Fixed Effects	Intercept	1		1
	time	2		1
	ICDcontrol	2		1
	time * ICDcontrol	4		1
Random Effects	Intercept[b]	1	Variance Components	1
Residual				1
Total		10		6

a. Dependent Variable: rsocf.

b. As of version 11.5, the syntax rules for the RANDOM subcommand have changed. Your command syntax may yield results that differ from those produced by prior versions. If you are using version 11 syntax, please consult the current syntax reference guide for more information.

Information Criteria[a]

-2 Restricted Log Likelihood	2554,689
Akaike's Information Criterion (AIC)	2558,689

Hurvich and Tsai's Criterion (AICC)	2558,733
Bozdogan's Criterion (CAIC)	2567,908
Schwarz's Bayesian Criterion (BIC)	2565,908

The information criteria are displayed in smaller-is-better form.

a. Dependent Variable: rsocf.

Fixed Effects

Type III Tests of Fixed Effects[a]

Source	Numerator df	Denominator df	F	Sig.
Intercept	1	,000	2198,742	,000
time	1	273	,015	,902
ICDcontrol	1	273	,712	,400
time * ICDcontrol	1	273	,069	,794

a. Dependent Variable: rsocf.

Estimates of Fixed Effects[a]

						95% Confidence Interval	
Parameter	Estimate	Std. Error	df	t	Sig.	Lower Bound	Upper Bound
Intercept	70,967742	3,207271	,000	22,127	,000	33,123561	108,811923
[time=1]	-1,176075	4,375440	273	-,269	,788	-9,789968	7,437817
[time=2]	0[b]	0	.	.	.	.	.
[ICDcontrol=1]	1,778160	4,554317	273	,390	,697	-7,187886	10,744205
[ICDcontrol=2]	0[b]	0	.	.	.	.	.
[time=1] * [ICDcontrol=1]	1,600905	6,113702	273	,262	,794	-10,435089	13,636900
[time=1] * [ICDcontrol=2]	0[b]	0	.	.	.	.	.
[time=2] * [ICDcontrol=1]	0[b]	0	.	.	.	.	.
[time=2] * [ICDcontrol=2]	0[b]	0	.	.	.	.	.

a. Dependent Variable: rsocf.

b. This parameter is set to zero because it is redundant.

Covariance Parameters

Estimates of Covariance Parameters[a]

Parameter		Estimate	Std. Error
Residual		637,768248	54,587923
Intercept	Variance	8,155777E-13	189812531,248503

a. Dependent Variable: rsocf.

Random Effect Covariance Structure (G)[a]

	Intercept
Intercept	8,155777E-13

Variance Components

a. Dependent Variable: rsocf.

Estimated Marginal Means

time * ICDcontrol[a]

time	ICDcontrol	Mean	Std. Error	df	95% Confidence Interval Lower Bound	95% Confidence Interval Upper Bound
1	Control	73,171	2,789	,000	40,643	105,699
	ICD	69,792	2,976	,000	34,370	105,213
2	Control	72,746	3,233	,000	37,515	107,977
	ICD	70,968	3,207	,000	33,124	108,812

a. Dependent Variable: rsocf.

```
TEMPORARY.
SELECT IF (ICDcontrol<=2 AND exclusie.x="Nee").
```

```
MIXED  rrolef BY time ICDcontrol
  /CRITERIA=CIN(95) MXITER(100) MXSTEP(10) SCORING(1) SINGULAR(0.000000000001) HCONVERGE(0,
    ABSOLUTE) LCONVERGE(0, ABSOLUTE) PCONVERGE(0.000001, ABSOLUTE)
  /FIXED=time ICDcontrol time*ICDcontrol | SSTYPE(3)
  /METHOD=REML
  /PRINT=DESCRIPTIVES G  SOLUTION
  /RANDOM=INTERCEPT | COVTYPE(VC)
  /EMMEANS=TABLES(time*ICDcontrol) .
```

Mixed Model Analysis

Notes

Output Created		23-AUG-2020 12:09:46
Comments		
Input	Data	M:\HP\Consult\HARTZ\Timal\ICD2\QOLlong.sav
	Active Dataset	DataSet3
	Filter	<none>
	Weight	<none>
	Split File	<none>
	N of Rows in Working Data File	342
Missing Value Handling	Definition of Missing	User-defined missing values are treated as missing.
	Cases Used	Statistics are based on all cases with valid data for all variables in the model.

Syntax		MIXED rrolef BY time ICDcontrol<br>/CRITERIA=CIN(95) MXITER(100) MXSTEP(10) SCORING(1) SINGULAR(0.000000000001) HCONVERGE(0, ABSOLUTE) LCONVERGE(0, ABSOLUTE) PCONVERGE(0.000001, ABSOLUTE)<br>/FIXED=time ICDcontrol time*ICDcontrol | SSTYPE(3)<br>/METHOD=REML<br>/PRINT=DESCRIPTIVES G SOLUTION<br>/RANDOM=INTERCEPT | COVTYPE(VC)<br><br>/EMMEANS=TABLES(time*ICDcontrol) .
Resources	Processor Time	00:00:00,05
	Elapsed Time	00:00:00,04

Warnings

The final Hessian matrix is not positive definite although all convergence criteria are satisfied. The MIXED procedure continues despite this warning. Validity of subsequent results cannot be ascertained.

Descriptive Statistics

rrolef

time	ICDcontrol	Count	Mean	Standard Deviation	Coefficient of Variation
1	Control	81	40,7407	41,16363	101,0%
	ICD	69	44,9275	43,84542	97,6%
	Total	150	42,6667	42,32610	99,2%
2	Control	59	33,4746	39,55849	118,2%

	ICD	59	51,6949	47,53955	92,0%
	Total	118	42,5847	44,49495	104,5%
Total	Control	140	37,6786	40,51117	107,5%
	ICD	128	48,0469	45,52941	94,8%
	Total	268	42,6306	43,21232	101,4%

Model Dimension[a]

		Number of Levels	Covariance Structure	Number of Parameters
Fixed Effects	Intercept	1		1
	time	2		1
	ICDcontrol	2		1
	time * ICDcontrol	4		1
Random Effects	Intercept[b]	1	Variance Components	1
Residual				1
Total		10		6

a. Dependent Variable: rrolef.

b. As of version 11.5, the syntax rules for the RANDOM subcommand have changed. Your command syntax may yield results that differ from those produced by prior versions. If you are using version 11 syntax, please consult the current syntax reference guide for more information.

Information Criteria[a]

-2 Restricted Log Likelihood	2751,890
Akaike's Information Criterion (AIC)	2755,890
Hurvich and Tsai's Criterion (AICC)	2755,936
Bozdogan's Criterion (CAIC)	2765,042
Schwarz's Bayesian Criterion (BIC)	2763,042

The information criteria are displayed in smaller-is-better form.

a. Dependent Variable: rrolef.

Fixed Effects

Type III Tests of Fixed Effects[a]

Source	Numerator df	Denominator df	F	Sig.
Intercept	1	264	259,891	,000
time	1	264	,002	,962
ICDcontrol	1	264	4,471	,035
time * ICDcontrol	1	264	1,754	,187

a. Dependent Variable: rrolef.

Estimates of Fixed Effects[a]

Parameter	Estimate	Std. Error	df	t	Sig.	95% Confidence Interval	
						Lower Bound	Upper Bound
Intercept	51,694915	5,598051	264	9,234	,000	40,672406	62,717424
[time=1]	-6,767379	7,624604	264	-,888	,376	-21,780152	8,245394
[time=2]	0[b]	0	.	.	.	.	.
[ICDcontrol=1]	-18,220339	7,916840	264	-2,301	,022	-33,808521	-2,632157
[ICDcontrol=2]	0[b]	0	.	.	.	.	.
[time=1] * [ICDcontrol=1]	14,033543	10,597138	264	1,324	,187	-6,832120	34,899207
[time=1] * [ICDcontrol=2]	0[b]	0	.	.	.	.	.
[time=2] * [ICDcontrol=1]	0[b]	0	.	.	.	.	.
[time=2] * [ICDcontrol=2]	0[b]	0	.	.	.	.	.

a. Dependent Variable: rrolef.

b. This parameter is set to zero because it is redundant.

Covariance Parameters

Estimates of Covariance Parameters[a]

Parameter		Estimate	Std. Error
Residual		1848,952324	160,930643
Intercept	Variance	,000000[b]	,000000

a. Dependent Variable: rrolef.

b. This covariance parameter is redundant.

Random Effect Covariance Structure (G)[a]

	Intercept
Intercept	,000000

Variance Components

a. Dependent Variable: rrolef.

Estimated Marginal Means

time * ICDcontrol[a]

time	ICDcontrol	Mean	Std. Error	df	95% Confidence Interval	
					Lower Bound	Upper Bound
1	Control	40,741	4,778	264	31,333	50,148
	ICD	44,928	5,177	264	34,735	55,120
2	Control	33,475	5,598	264	22,452	44,497
	ICD	51,695	5,598	264	40,672	62,717

a. Dependent Variable: rrolef.

```
TEMPORARY.
SELECT IF (ICDcontrol<=2 AND exclusie.x="Nee").
MIXED rrole BY time ICDcontrol
  /CRITERIA=CIN(95) MXITER(100) MXSTEP(10) SCORING(1) SINGULAR(0.000000000001) HCONVERGE(0,
    ABSOLUTE) LCONVERGE(0, ABSOLUTE) PCONVERGE(0.000001, ABSOLUTE)
  /FIXED=time ICDcontrol time*ICDcontrol | SSTYPE(3)
  /METHOD=REML
  /PRINT=DESCRIPTIVES G  SOLUTION
  /RANDOM=INTERCEPT | COVTYPE(VC)
  /EMMEANS=TABLES(time*ICDcontrol) .
```

Mixed Model Analysis

Notes

Output Created		23-AUG-2020 12:09:46
Comments		
Input	Data	M:\HP\Consult\HARTZ\Timal\ICD2\QOLlong.sav
	Active Dataset	DataSet3
	Filter	<none>
	Weight	<none>
	Split File	<none>
	N of Rows in Working Data File	342
Missing Value Handling	Definition of Missing	User-defined missing values are treated as missing.
	Cases Used	Statistics are based on all cases with valid data for all variables in the model.
Syntax		MIXED rrole BY time ICDcontrol /CRITERIA=CIN(95) MXITER(100) MXSTEP(10) SCORING(1) SINGULAR(0.000000000001) HCONVERGE(0, ABSOLUTE) LCONVERGE(0, ABSOLUTE) PCONVERGE(0.000001, ABSOLUTE) /FIXED=time ICDcontrol time*ICDcontrol | SSTYPE(3) /METHOD=REML /PRINT=DESCRIPTIVES G SOLUTION /RANDOM=INTERCEPT | COVTYPE(VC) /EMMEANS=TABLES(time*ICDcontrol) .
Resources	Processor Time	00:00:00,06

Elapsed Time	00:00:00,07

Descriptive Statistics

rrole

time	ICDcontrol	Count	Mean	Standard Deviation	Coefficient of Variation
1	Control	79	72,1519	37,90466	52,5%
	ICD	67	68,1592	43,20215	63,4%
	Total	146	70,3196	40,32878	57,4%
2	Control	59	65,5367	41,50710	63,3%
	ICD	60	72,2222	42,59327	59,0%
	Total	119	68,9076	42,01405	61,0%
Total	Control	138	69,3237	39,47375	56,9%
	ICD	127	70,0787	42,79368	61,1%
	Total	265	69,6855	41,02159	58,9%

Model Dimension[a]

		Number of Levels	Covariance Structure	Number of Parameters
Fixed Effects	Intercept	1		1
	time	2		1
	ICDcontrol	2		1
	time * ICDcontrol	4		1
Random Effects	Intercept[b]	1	Variance Components	1
Residual				1
Total		10		6

a. Dependent Variable: rrole.

b. As of version 11.5, the syntax rules for the RANDOM subcommand have changed. Your command syntax may yield results that differ from those produced by prior versions. If you are using version 11 syntax, please consult the current syntax reference guide for more information.

Information Criteria[a]

-2 Restricted Log Likelihood	2697,974

Akaike's Information Criterion (AIC)	2701,974
Hurvich and Tsai's Criterion (AICC)	2702,021
Bozdogan's Criterion (CAIC)	2711,103
Schwarz's Bayesian Criterion (BIC)	2709,103

The information criteria are displayed in smaller-is-better form.

a. Dependent Variable: rrole.

Fixed Effects

Type III Tests of Fixed Effects[a]

Source	Numerator df	Denominator df	F	Sig.
Intercept	1	,000	5,661	,000
time	1	261,000	,063	,802
ICDcontrol	1	261,000	,070	,792
time * ICDcontrol	1	261,000	1,100	,295

a. Dependent Variable: rrole.

Estimates of Fixed Effects[a]

Parameter	Estimate	Std. Error	df	t	Sig.	95% Confidence Interval	
						Lower Bound	Upper Bound
Intercept	72,222222	29,586996	,000	2,441	,000	,119782	144,324663
[time=1]	-4,063018	7,316182	261,000	-,555	,579	-18,469273	10,343236
[time=2]	0[b]	0	.	.	.	.	.
[ICDcontrol=1]	-6,685499	7,546881	261,000	-,886	,377	-21,546022	8,175024
[ICDcontrol=2]	0[b]	0	.	.	.	.	.
[time=1] * [ICDcontrol=1]	10,678194	10,182849	261,000	1,049	,295	-9,372800	30,729188
[time=1] * [ICDcontrol=2]	0[b]	0	.	.	.	.	.
[time=2] * [ICDcontrol=1]	0[b]	0	.	.	.	.	.
[time=2] * [ICDcontrol=2]	0[b]	0	.	.	.	.	.

a. Dependent Variable: rrole.

b. This parameter is set to zero because it is redundant.

Covariance Parameters

Estimates of Covariance Parameters[a]

Parameter		Estimate	Std. Error
Residual		1694,303822	148,315327
Intercept	Variance	847,151911	68719476735,999990

a. Dependent Variable: rrole.

Random Effect Covariance Structure (G)[a]

	Intercept
Intercept	847,151911

Variance Components

a. Dependent Variable: rrole.

Estimated Marginal Means

time * ICDcontrol[a]

time	ICDcontrol	Mean	Std. Error	df	95% Confidence Interval Lower Bound	95% Confidence Interval Upper Bound
1	Control	72,152	29,472	,000	-13,821	158,124
	ICD	68,159	29,537	,000	-9,614	145,932
2	Control	65,537	29,595	,000	-22,857	153,931
	ICD	72,222	29,587	,000	,120	144,325

a. Dependent Variable: rrole.

```
TEMPORARY.
SELECT IF (ICDcontrol<=2 AND exclusie.x="Nee").
MIXED rment BY time ICDcontrol
  /CRITERIA=CIN(95) MXITER(100) MXSTEP(10) SCORING(1) SINGULAR(0.000000000001) HCONVERGE(0,
    ABSOLUTE) LCONVERGE(0, ABSOLUTE) PCONVERGE(0.000001, ABSOLUTE)
  /FIXED=time ICDcontrol time*ICDcontrol | SSTYPE(3)
  /METHOD=REML
  /PRINT=DESCRIPTIVES G  SOLUTION
  /RANDOM=INTERCEPT | COVTYPE(VC)
  /EMMEANS=TABLES(time*ICDcontrol) .
```

Mixed Model Analysis

Notes

Output Created		23-AUG-2020 12:09:46
Comments		
Input	Data	M:\HP\Consult\HARTZ\Timal\ICD2\QOLlong.sav
	Active Dataset	DataSet3
	Filter	<none>
	Weight	<none>
	Split File	<none>
	N of Rows in Working Data File	342
Missing Value Handling	Definition of Missing	User-defined missing values are treated as missing.
	Cases Used	Statistics are based on all cases with valid data for all variables in the model.

Syntax		MIXED rment BY time ICDcontrol<br>/CRITERIA=CIN(95) MXITER(100) MXSTEP(10) SCORING(1) SINGULAR(0.000000000001) HCONVERGE(0,<br>ABSOLUTE) LCONVERGE(0, ABSOLUTE) PCONVERGE(0.000001, ABSOLUTE)<br>/FIXED=time ICDcontrol time*ICDcontrol | SSTYPE(3)<br>/METHOD=REML<br>/PRINT=DESCRIPTIVES G SOLUTION<br>/RANDOM=INTERCEPT | COVTYPE(VC)<br><br>/EMMEANS=TABLES(time*ICDcontrol) .
Resources	Processor Time	00:00:00,03
	Elapsed Time	00:00:00,04

Warnings

The final Hessian matrix is not positive definite although all convergence criteria are satisfied. The MIXED procedure continues despite this warning. Validity of subsequent results cannot be ascertained.

Descriptive Statistics

rment

time	ICDcontrol	Count	Mean	Standard Deviation	Coefficient of Variation
1	Control	82	79,1220	15,31915	19,4%
	ICD	72	76,5000	17,15464	22,4%
	Total	154	77,8961	16,20263	20,8%
2	Control	60	77,8667	15,76895	20,3%

	ICD	62	77,6129	15,77647	20,3%
	Total	122	77,7377	15,70798	20,2%
Total	Control	142	78,5915	15,46772	19,7%
	ICD	134	77,0149	16,47919	21,4%
	Total	276	77,8261	15,95718	20,5%

Model Dimension[a]

		Number of Levels	Covariance Structure	Number of Parameters
Fixed Effects	Intercept	1		1
	time	2		1
	ICDcontrol	2		1
	time * ICDcontrol	4		1
Random Effects	Intercept[b]	1	Variance Components	1
Residual				1
Total		10		6

a. Dependent Variable: rment.

b. As of version 11.5, the syntax rules for the RANDOM subcommand have changed. Your command syntax may yield results that differ from those produced by prior versions. If you are using version 11 syntax, please consult the current syntax reference guide for more information.

Information Criteria[a]

-2 Restricted Log Likelihood	2297,581
Akaike's Information Criterion (AIC)	2301,581
Hurvich and Tsai's Criterion (AICC)	2301,626
Bozdogan's Criterion (CAIC)	2310,793
Schwarz's Bayesian Criterion (BIC)	2308,793

The information criteria are displayed in smaller-is-better form.

a. Dependent Variable: rment.

Fixed Effects

Type III Tests of Fixed Effects[a]

Source	Numerator df	Denominator df	F	Sig.
Intercept	1	272,000	6409,488	,000
time	1	272,000	,001	,971
ICDcontrol	1	272,000	,548	,460
time * ICDcontrol	1	272,000	,371	,543

a. Dependent Variable: rment.

Estimates of Fixed Effects[a]

Parameter	Estimate	Std. Error	df	t	Sig.	95% Confidence Interval	
						Lower Bound	Upper Bound
Intercept	77,612903	2,033817	272,000	38,161	,000	73,608880	81,616927
[time=1]	-1,112903	2,774585	272,000	-,401	,689	-6,575294	4,349487
[time=2]	0[b]	0	.	.	.	.	.
[ICDcontrol=1]	,253763	2,900121	272,000	,088	,930	-5,455774	5,963301
[ICDcontrol=2]	0[b]	0	.	.	.	.	.
[time=1] * [ICDcontrol=1]	2,368188	3,885890	272,000	,609	,543	-5,282056	10,018432
[time=1] * [ICDcontrol=2]	0[b]	0	.	.	.	.	.
[time=2] * [ICDcontrol=1]	0[b]	0	.	.	.	.	.
[time=2] * [ICDcontrol=2]	0[b]	0	.	.	.	.	.

a. Dependent Variable: rment.

b. This parameter is set to zero because it is redundant.

Covariance Parameters

Estimates of Covariance Parameters[a]

Parameter		Estimate	Std. Error
Residual		256,457439	21,991044
Intercept	Variance	,000000[b]	,000000

a. Dependent Variable: rment.

b. This covariance parameter is redundant.

Random Effect Covariance Structure (G)[a]

	Intercept
Intercept	,000000

Variance Components

a. Dependent Variable: rment.

Estimated Marginal Means

time * ICDcontrol[a]

time	ICDcontrol	Mean	Std. Error	df	95% Confidence Interval	
					Lower Bound	Upper Bound
1	Control	79,122	1,768	272,000	75,640	82,604
	ICD	76,500	1,887	272,000	72,784	80,216
2	Control	77,867	2,067	272,000	73,796	81,937
	ICD	77,613	2,034	272,000	73,609	81,617

a. Dependent Variable: rment.

```
TEMPORARY.
SELECT IF (ICDcontrol<=2 AND exclusie.x="Nee").
MIXED rvit BY time ICDcontrol
  /CRITERIA=CIN(95) MXITER(100) MXSTEP(10) SCORING(1) SINGULAR(0.000000000001) HCONVERGE(0,
    ABSOLUTE) LCONVERGE(0, ABSOLUTE) PCONVERGE(0.000001, ABSOLUTE)
  /FIXED=time ICDcontrol time*ICDcontrol | SSTYPE(3)
  /METHOD=REML
  /PRINT=DESCRIPTIVES G  SOLUTION
  /RANDOM=INTERCEPT | COVTYPE(VC)
  /EMMEANS=TABLES(time*ICDcontrol) .
```

Mixed Model Analysis

Notes

Output Created		23-AUG-2020 12:09:47
Comments		
Input	Data	M:\HP\Consult\HARTZ\Timal\ICD2\QOLlong.sav
	Active Dataset	DataSet3
	Filter	<none>
	Weight	<none>
	Split File	<none>
	N of Rows in Working Data File	342
Missing Value Handling	Definition of Missing	User-defined missing values are treated as missing.
	Cases Used	Statistics are based on all cases with valid data for all variables in the model.
Syntax		MIXED rvit BY time ICDcontrol /CRITERIA=CIN(95) MXITER(100) MXSTEP(10) SCORING(1) SINGULAR(0.000000000001) HCONVERGE(0, ABSOLUTE) LCONVERGE(0, ABSOLUTE) PCONVERGE(0.000001, ABSOLUTE) /FIXED=time ICDcontrol time*ICDcontrol | SSTYPE(3) /METHOD=REML /PRINT=DESCRIPTIVES G SOLUTION /RANDOM=INTERCEPT | COVTYPE(VC) /EMMEANS=TABLES(time*ICDcontrol) .
Resources	Processor Time	00:00:00,05

Elapsed Time	00:00:00,05

Descriptive Statistics

rvit

time	ICDcontrol	Count	Mean	Standard Deviation	Coefficient of Variation
1	Control	82	57,1341	17,98543	31,5%
	ICD	72	54,0972	20,91779	38,7%
	Total	154	55,7143	19,40646	34,8%
2	Control	60	50,6667	20,26240	40,0%
	ICD	62	56,6129	22,48450	39,7%
	Total	122	53,6885	21,53992	40,1%
Total	Control	142	54,4014	19,18076	35,3%
	ICD	134	55,2612	21,61103	39,1%
	Total	276	54,8188	20,36405	37,1%

Model Dimension[a]

		Number of Levels	Covariance Structure	Number of Parameters
Fixed Effects	Intercept	1		1
	time	2		1
	ICDcontrol	2		1
	time * ICDcontrol	4		1
Random Effects	Intercept[b]	1	Variance Components	1
Residual				1
Total		10		6

a. Dependent Variable: rvit.

b. As of version 11.5, the syntax rules for the RANDOM subcommand have changed. Your command syntax may yield results that differ from those produced by prior versions. If you are using version 11 syntax, please consult the current syntax reference guide for more information.

Information Criteria[a]

-2 Restricted Log Likelihood	2427,170

Akaike's Information Criterion (AIC)	2431,170
Hurvich and Tsai's Criterion (AICC)	2431,215
Bozdogan's Criterion (CAIC)	2440,382
Schwarz's Bayesian Criterion (BIC)	2438,382

The information criteria are displayed in smaller-is-better form.

a. Dependent Variable: rvit.

Fixed Effects

Type III Tests of Fixed Effects[a]

Source	Numerator df	Denominator df	F	Sig.
Intercept	1	,000	238,359	,000
time	1	272	,642	,424
ICDcontrol	1	272	,348	,556
time * ICDcontrol	1	272	3,319	,070

a. Dependent Variable: rvit.

Estimates of Fixed Effects[a]

Parameter	Estimate	Std. Error	df	t	Sig.	95% Confidence Interval	
						Lower Bound	Upper Bound
Intercept	56,612903	4,202491	,000	13,471	,000	35,339140	77,886667
[time=1]	-2,515681	3,520899	272	-,714	,476	-9,447358	4,415996
[time=2]	0[b]	0	.	.	.	.	.
[ICDcontrol=1]	-5,946237	3,680202	272	-1,616	,107	-13,191538	1,299065
[ICDcontrol=2]	0[b]	0	.	.	.	.	.
[time=1] * [ICDcontrol=1]	8,983161	4,931125	272	1,822	,070	-,724863	18,691185
[time=1] * [ICDcontrol=2]	0[b]	0	.	.	.	.	.
[time=2] * [ICDcontrol=1]	0[b]	0	.	.	.	.	.
[time=2] * [ICDcontrol=2]	0[b]	0	.	.	.	.	.

a. Dependent Variable: rvit.

b. This parameter is set to zero because it is redundant.

Covariance Parameters

Estimates of Covariance Parameters[a]

Parameter		Estimate	Std. Error
Residual		412,977525	35,412531
Intercept	Variance	11,000000	1073741824,000000

a. Dependent Variable: rvit.

Random Effect Covariance Structure (G)[a]

	Intercept
Intercept	11,000000

Variance Components

a. Dependent Variable: rvit.

Estimated Marginal Means

time * ICDcontrol[a]

time	ICDcontrol	Mean	Std. Error	df	95% Confidence Interval	
					Lower Bound	Upper Bound
1	Control	57,134	4,005	,000	35,019	79,249
	ICD	54,097	4,091	,000	39,023	69,171
2	Control	50,667	4,229	,000	36,346	64,987
	ICD	56,613	4,202	,000	35,339	77,887

a. Dependent Variable: rvit.

```
TEMPORARY.
SELECT IF (ICDcontrol<=2 AND exclusie.x="Nee").
MIXED rpain BY time ICDcontrol
  /CRITERIA=CIN(95) MXITER(100) MXSTEP(10) SCORING(1) SINGULAR(0.000000000001) HCONVERGE(0,
    ABSOLUTE) LCONVERGE(0, ABSOLUTE) PCONVERGE(0.000001, ABSOLUTE)
  /FIXED=time ICDcontrol time*ICDcontrol | SSTYPE(3)
  /METHOD=REML
  /PRINT=DESCRIPTIVES G  SOLUTION
  /RANDOM=INTERCEPT | COVTYPE(VC)
  /EMMEANS=TABLES(time*ICDcontrol) .
```

Mixed Model Analysis

Notes

Output Created		23-AUG-2020 12:09:47
Comments		
Input	Data	M:\HP\Consult\HARTZ\Timal\ICD2\QOLlong.sav
	Active Dataset	DataSet3
	Filter	<none>
	Weight	<none>
	Split File	<none>
	N of Rows in Working Data File	342
Missing Value Handling	Definition of Missing	User-defined missing values are treated as missing.
	Cases Used	Statistics are based on all cases with valid data for all variables in the model.

Syntax		MIXED rpain BY time ICDcontrol<br>/CRITERIA=CIN(95)<br>MXITER(100) MXSTEP(10)<br>SCORING(1)<br>SINGULAR(0.000000000001)<br>HCONVERGE(0,<br>ABSOLUTE) LCONVERGE(0,<br>ABSOLUTE)<br>PCONVERGE(0.000001,<br>ABSOLUTE)<br>/FIXED=time ICDcontrol<br>time*ICDcontrol | SSTYPE(3)<br>/METHOD=REML<br>/PRINT=DESCRIPTIVES G<br>SOLUTION<br>/RANDOM=INTERCEPT | COVTYPE(VC)<br><br>/EMMEANS=TABLES(time*ICDc<br>ontrol) .
Resources	Processor Time	00:00:00,05
	Elapsed Time	00:00:00,04

Descriptive Statistics

rpain

time	ICDcontrol	Count	Mean	Standard Deviation	Coefficient of Variation
1	Control	82	73,4881	23,62041	32,1%
	ICD	72	73,5473	22,56753	30,7%
	Total	154	73,5158	23,05886	31,4%
2	Control	61	71,4420	24,99390	35,0%
	ICD	62	70,4411	30,33046	43,1%
	Total	123	70,9374	27,70285	39,1%
Total	Control	143	72,6152	24,15035	33,3%
	ICD	134	72,1101	26,38600	36,6%
	Total	277	72,3709	25,21179	34,8%

Model Dimension[a]

		Number of Levels	Covariance Structure	Number of Parameters
Fixed Effects	Intercept	1		1
	time	2		1
	ICDcontrol	2		1
	time * ICDcontrol	4		1
Random Effects	Intercept[b]	1	Variance Components	1
Residual				1
Total		10		6

a. Dependent Variable: rpain.

b. As of version 11.5, the syntax rules for the RANDOM subcommand have changed. Your command syntax may yield results that differ from those produced by prior versions. If you are using version 11 syntax, please consult the current syntax reference guide for more information.

Information Criteria[a]

-2 Restricted Log Likelihood	2556,001
Akaike's Information Criterion (AIC)	2560,001
Hurvich and Tsai's Criterion (AICC)	2560,045
Bozdogan's Criterion (CAIC)	2569,220
Schwarz's Bayesian Criterion (BIC)	2567,220

The information criteria are displayed in smaller-is-better form.

a. Dependent Variable: rpain.

Fixed Effects

Type III Tests of Fixed Effects[a]

Source	Numerator df	Denominator df	F	Sig.

Intercept	1	,000	1200,071	,000
time	1	273	,707	,401
ICDcontrol	1	273	,024	,878
time * ICDcontrol	1	273	,030	,863

a. Dependent Variable: rpain.

Estimates of Fixed Effects[a]

Parameter	Estimate	Std. Error	df	t	Sig.	95% Confidence Interval	
						Lower Bound	Upper Bound
Intercept	70,441080	3,512284	,000	20,056	,000	-10,715915	151,598074
[time=1]	3,106256	4,385967	273	,708	,479	-5,528360	11,740872
[time=2]	0[b]	0	.	.	.	.	.
[ICDcontrol=1]	1,000874	4,565274	273	,219	,827	-7,986742	9,988490
[ICDcontrol=2]	0[b]	0	.	.	.	.	.
[time=1] * [ICDcontrol=1]	-1,060156	6,128411	273	-,173	,863	-13,125107	11,004795
[time=1] * [ICDcontrol=2]	0[b]	0	.	.	.	.	.
[time=2] * [ICDcontrol=1]	0[b]	0	.	.	.	.	.
[time=2] * [ICDcontrol=2]	0[b]	0	.	.	.	.	.

a. Dependent Variable: rpain.

b. This parameter is set to zero because it is redundant.

Covariance Parameters

Estimates of Covariance Parameters[a]

Parameter		Estimate	Std. Error
Residual		640,840650	54,850896
Intercept	Variance	2,000000	309962565,56328<br>0

a. Dependent Variable: rpain.

Random Effect Covariance Structure (G)[a]

	Intercept
Intercept	2,000000

Variance Components

a. Dependent Variable: rpain.

Estimated Marginal Means

time * ICDcontrol[a]

time	ICDcontrol	Mean	Std. Error	df	95% Confidence Interval	
					Lower Bound	Upper Bound
1	Control	73,488	3,133	,000	51,362	95,614
	ICD	73,547	3,302	,000	48,405	98,689
2	Control	71,442	3,536	,000	20,133	122,751
	ICD	70,441	3,512	,000	-10,716	151,598

a. Dependent Variable: rpain.

```
TEMPORARY.
SELECT IF (ICDcontrol<=2 AND exclusie.x="Nee").
MIXED rgenh BY time ICDcontrol
  /CRITERIA=CIN(95) MXITER(100) MXSTEP(10) SCORING(1) SINGULAR(0.000000000001) HCONVERGE(0,
    ABSOLUTE) LCONVERGE(0, ABSOLUTE) PCONVERGE(0.000001, ABSOLUTE)
  /FIXED=time ICDcontrol time*ICDcontrol | SSTYPE(3)
  /METHOD=REML
  /PRINT=DESCRIPTIVES G  SOLUTION
  /RANDOM=INTERCEPT | COVTYPE(VC)
  /EMMEANS=TABLES(time*ICDcontrol) .
```

Mixed Model Analysis

Notes

Output Created		23-AUG-2020 12:09:47
Comments		
Input	Data	M:\HP\Consult\HARTZ\Timal\ICD2\QOLlong.sav
	Active Dataset	DataSet3
	Filter	<none>
	Weight	<none>
	Split File	<none>
	N of Rows in Working Data File	342
Missing Value Handling	Definition of Missing	User-defined missing values are treated as missing.
	Cases Used	Statistics are based on all cases with valid data for all variables in the model.
Syntax		MIXED rgenh BY time ICDcontrol<br>/CRITERIA=CIN(95) MXITER(100) MXSTEP(10) SCORING(1) SINGULAR(0.000000000001) HCONVERGE(0,<br>ABSOLUTE) LCONVERGE(0, ABSOLUTE) PCONVERGE(0.000001, ABSOLUTE)<br>/FIXED=time ICDcontrol time*ICDcontrol | SSTYPE(3)<br>/METHOD=REML<br>/PRINT=DESCRIPTIVES G SOLUTION<br>/RANDOM=INTERCEPT | COVTYPE(VC)<br><br>/EMMEANS=TABLES(time*ICDcontrol) .
Resources	Processor Time	00:00:00,03
	Elapsed Time	00:00:00,04

Descriptive Statistics

rgenh

time	ICDcontrol	Count	Mean	Standard Deviation	Coefficient of Variation
1	Control	82	42,9878	17,60085	40,9%
	ICD	70	40,7143	18,71382	46,0%
	Total	152	41,9408	18,09696	43,1%
2	Control	61	42,0492	19,67183	46,8%
	ICD	60	42,0833	16,26819	38,7%
	Total	121	42,0661	17,98923	42,8%
Total	Control	143	42,5874	18,45105	43,3%
	ICD	130	41,3462	17,57364	42,5%
	Total	273	41,9963	18,01623	42,9%

Model Dimension[a]

		Number of Levels	Covariance Structure	Number of Parameters
Fixed Effects	Intercept	1		1
	time	2		1
	ICDcontrol	2		1
	time * ICDcontrol	4		1
Random Effects	Intercept[b]	1	Variance Components	1
Residual				1
Total		10		6

a. Dependent Variable: rgenh.

b. As of version 11.5, the syntax rules for the RANDOM subcommand have changed. Your command syntax may yield results that differ from those produced by prior versions. If you are using version 11 syntax, please consult the current syntax reference guide for more information.

Information Criteria[a]

-2 Restricted Log Likelihood	2338,139
Akaike's Information Criterion (AIC)	2342,139

Hurvich and Tsai's Criterion (AICC)	2342,184
Bozdogan's Criterion (CAIC)	2351,328
Schwarz's Bayesian Criterion (BIC)	2349,328

The information criteria are displayed in smaller-is-better form.

a. Dependent Variable: rgenh.

Fixed Effects

Type III Tests of Fixed Effects[a]

Source	Numerator df	Denominator df	F	Sig.
Intercept	1	,000	10,673	,000
time	1	269,000	,010	,922
ICDcontrol	1	269,000	,257	,612
time * ICDcontrol	1	269,000	,273	,602

a. Dependent Variable: rgenh.

Estimates of Fixed Effects[a]

Parameter	Estimate	Std. Error	df	t	Sig.	95% Confidence Interval	
						Lower Bound	Upper Bound
Intercept	42,083333	13,007509	,000	3,235	,000	11,452226	72,714441
[time=1]	-1,369048	3,183728	269,000	-,430	,668	-7,637241	4,899146
[time=2]	0[b]	0	.	.	.	.	.
[ICDcontrol=1]	-,034153	3,290342	269,000	-,010	,992	-6,512251	6,443945
[ICDcontrol=2]	0[b]	0	.	.	.	.	.
[time=1] * [ICDcontrol=1]	2,307672	4,415672	269,000	,523	,602	-6,385999	11,001344
[time=1] * [ICDcontrol=2]	0[b]	0	.	.	.	.	.
[time=2] * [ICDcontrol=1]	0[b]	0	.	.	.	.	.
[time=2] * [ICDcontrol=2]	0[b]	0	.	.	.	.	.

a. Dependent Variable: rgenh.

b. This parameter is set to zero because it is redundant.

Covariance Parameters

Estimates of Covariance Parameters[a]

Parameter		Estimate	Std. Error
Residual		327,474756	28,236878
Intercept	Variance	163,737378	17179869184,000380

a. Dependent Variable: rgenh.

Random Effect Covariance Structure (G)[a]

	Intercept
Intercept	163,737378

Variance Components

a. Dependent Variable: rgenh.

Estimated Marginal Means

time * ICDcontrol[a]

					95% Confidence Interval	
time	ICDcontrol	Mean	Std. Error	df	Lower Bound	Upper Bound
1	Control	42,988	12,951	,000	17,662	68,314
	ICD	40,714	12,978	,000	5,504	75,925
2	Control	42,049	13,004	,000	10,931	73,168
	ICD	42,083	13,008	,000	11,452	72,714

a. Dependent Variable: rgenh.

```
TEMPORARY.
SELECT IF (ICDcontrol<=2 AND exclusie.x="Nee").
```

```
MIXED rhchange BY time ICDcontrol
  /CRITERIA=CIN(95) MXITER(100) MXSTEP(10) SCORING(1) SINGULAR(0.000000000001) HCONVERGE(0,
    ABSOLUTE) LCONVERGE(0, ABSOLUTE) PCONVERGE(0.000001, ABSOLUTE)
  /FIXED=time ICDcontrol time*ICDcontrol | SSTYPE(3)
  /METHOD=REML
  /PRINT=DESCRIPTIVES G  SOLUTION
  /RANDOM=INTERCEPT | COVTYPE(VC)
  /EMMEANS=TABLES(time*ICDcontrol) .
```

Mixed Model Analysis

Notes

Output Created		23-AUG-2020 12:09:47
Comments		
Input	Data	M:\HP\Consult\HARTZ\Timal\ICD2\QOLlong.sav
	Active Dataset	DataSet3
	Filter	<none>
	Weight	<none>
	Split File	<none>
	N of Rows in Working Data File	342
Missing Value Handling	Definition of Missing	User-defined missing values are treated as missing.
	Cases Used	Statistics are based on all cases with valid data for all variables in the model.

Syntax		MIXED rhchange BY time ICDcontrol<br>/CRITERIA=CIN(95) MXITER(100) MXSTEP(10) SCORING(1) SINGULAR(0.000000000001) HCONVERGE(0,<br>ABSOLUTE) LCONVERGE(0, ABSOLUTE) PCONVERGE(0.000001, ABSOLUTE)<br>/FIXED=time ICDcontrol time*ICDcontrol | SSTYPE(3)<br>/METHOD=REML<br>/PRINT=DESCRIPTIVES G SOLUTION<br>/RANDOM=INTERCEPT | COVTYPE(VC)<br><br>/EMMEANS=TABLES(time*ICDcontrol) .
Resources	Processor Time	00:00:00,03
	Elapsed Time	00:00:00,04

Warnings

The final Hessian matrix is not positive definite although all convergence criteria are satisfied. The MIXED procedure continues despite this warning. Validity of subsequent results cannot be ascertained.

Descriptive Statistics

rhchange

time	ICDcontrol	Count	Mean	Standard Deviation	Coefficient of Variation
1	Control	82	59,4512	27,11437	45,6%
	ICD	72	59,7222	30,99169	51,9%
	Total	154	59,5779	28,89553	48,5%
2	Control	61	48,7705	24,33543	49,9%

	ICD	62	56,0484	24,24494	43,3%
	Total	123	52,4390	24,46448	46,7%
Total	Control	143	54,8951	26,41401	48,1%
	ICD	134	58,0224	28,03072	48,3%
	Total	277	56,4079	27,20367	48,2%

Model Dimension[a]

		Number of Levels	Covariance Structure	Number of Parameters
Fixed Effects	Intercept	1		1
	time	2		1
	ICDcontrol	2		1
	time * ICDcontrol	4		1
Random Effects	Intercept[b]	1	Variance Components	1
Residual				1
Total		10		6

a. Dependent Variable: rhchange.

b. As of version 11.5, the syntax rules for the RANDOM subcommand have changed. Your command syntax may yield results that differ from those produced by prior versions. If you are using version 11 syntax, please consult the current syntax reference guide for more information.

Information Criteria[a]

-2 Restricted Log Likelihood	2591,350
Akaike's Information Criterion (AIC)	2595,350
Hurvich and Tsai's Criterion (AICC)	2595,394
Bozdogan's Criterion (CAIC)	2604,569
Schwarz's Bayesian Criterion (BIC)	2602,569

The information criteria are displayed in smaller-is-better form.

a. Dependent Variable: rhchange.

Fixed Effects

Type III Tests of Fixed Effects[a]

Source	Numerator df	Denominator df	F	Sig.
Intercept	1	273,000	1173,646	,000
time	1	273	4,820	,029
ICDcontrol	1	273	1,333	,249
time * ICDcontrol	1	273	1,148	,285

a. Dependent Variable: rhchange.

Estimates of Fixed Effects[a]

Parameter	Estimate	Std. Error	df	t	Sig.	95% Confidence Interval	
						Lower Bound	Upper Bound
Intercept	56,048387	3,430015	273	16,341	,000	49,295746	62,801028
[time=1]	3,673835	4,679313	273	,785	,433	-5,538290	12,885960
[time=2]	0[b]	0	.	.	.	.	.
[ICDcontrol=1]	-7,277895	4,870613	273	-1,494	,136	-16,866630	2,310839
[ICDcontrol=2]	0[b]	0	.	.	.	.	.
[time=1] * [ICDcontrol=1]	7,006893	6,538297	273	1,072	,285	-5,864998	19,878783
[time=1] * [ICDcontrol=2]	0[b]	0	.	.	.	.	.
[time=2] * [ICDcontrol=1]	0[b]	0	.	.	.	.	.
[time=2] * [ICDcontrol=2]	0[b]	0	.	.	.	.	.

a. Dependent Variable: rhchange.

b. This parameter is set to zero because it is redundant.

Covariance Parameters

Estimates of Covariance Parameters[a]

Parameter		Estimate	Std. Error
Residual		729,430004	62,433445
Intercept	Variance	,000000[b]	,000000

a. Dependent Variable: rhchange.

b. This covariance parameter is redundant.

Random Effect Covariance Structure (G)[a]

	Intercept
Intercept	,000000

Variance Components

a. Dependent Variable: rhchange.

Estimated Marginal Means

time * ICDcontrol[a]

time	ICDcontrol	Mean	Std. Error	df	95% Confidence Interval Lower Bound	95% Confidence Interval Upper Bound
1	Control	59,451	2,983	273	53,580	65,323
	ICD	59,722	3,183	273	53,456	65,988
2	Control	48,770	3,458	273	41,963	55,578
	ICD	56,048	3,430	273	49,296	62,801

a. Dependent Variable: rhchange.

```
TEMPORARY.
SELECT IF (ICDcontrol<=2 AND exclusie.x="Nee").
MIXED hvas BY time ICDcontrol
  /CRITERIA=CIN(95) MXITER(100) MXSTEP(10) SCORING(1) SINGULAR(0.000000000001) HCONVERGE(0,
    ABSOLUTE) LCONVERGE(0, ABSOLUTE) PCONVERGE(0.000001, ABSOLUTE)
  /FIXED=time ICDcontrol time*ICDcontrol | SSTYPE(3)
  /METHOD=REML
  /PRINT=DESCRIPTIVES G  SOLUTION
  /RANDOM=INTERCEPT | COVTYPE(VC)
  /EMMEANS=TABLES(time*ICDcontrol) .
```

Mixed Model Analysis

Notes

Output Created		23-AUG-2020 12:09:47
Comments		
Input	Data	M:\HP\Consult\HARTZ\Timal\ICD2\QOLlong.sav
	Active Dataset	DataSet3
	Filter	<none>
	Weight	<none>
	Split File	<none>
	N of Rows in Working Data File	342
Missing Value Handling	Definition of Missing	User-defined missing values are treated as missing.
	Cases Used	Statistics are based on all cases with valid data for all variables in the model.
Syntax		MIXED hvas BY time ICDcontrol /CRITERIA=CIN(95) MXITER(100) MXSTEP(10) SCORING(1) SINGULAR(0.000000000001) HCONVERGE(0, ABSOLUTE) LCONVERGE(0, ABSOLUTE) PCONVERGE(0.000001, ABSOLUTE) /FIXED=time ICDcontrol time*ICDcontrol | SSTYPE(3) /METHOD=REML /PRINT=DESCRIPTIVES G SOLUTION /RANDOM=INTERCEPT | COVTYPE(VC) /EMMEANS=TABLES(time*ICDcontrol) .
Resources	Processor Time	00:00:00,03

Elapsed Time	00:00:00,03

Descriptive Statistics

hvas

time	ICDcontrol	Count	Mean	Standard Deviation	Coefficient of Variation
1	Control	77	62,9221	21,35771	33,9%
	ICD	70	62,1143	20,30602	32,7%
	Total	147	62,5374	20,79623	33,3%
2	Control	61	62,8525	19,78875	31,5%
	ICD	61	61,0984	20,66213	33,8%
	Total	122	61,9754	20,16562	32,5%
Total	Control	138	62,8913	20,60461	32,8%
	ICD	131	61,6412	20,39988	33,1%
	Total	269	62,2825	20,47646	32,9%

Model Dimension[a]

		Number of Levels	Covariance Structure	Number of Parameters
Fixed Effects	Intercept	1		1
	time	2		1
	ICDcontrol	2		1
	time * ICDcontrol	4		1
Random Effects	Intercept[b]	1	Variance Components	1
Residual				1
Total		10		6

a. Dependent Variable: hvas.

b. As of version 11.5, the syntax rules for the RANDOM subcommand have changed. Your command syntax may yield results that differ from those produced by prior versions. If you are using version 11 syntax, please consult the current syntax reference guide for more information.

Information Criteria[a]

-2 Restricted Log Likelihood	2371,723

Akaike's Information Criterion (AIC)	2375,723
Hurvich and Tsai's Criterion (AICC)	2375,769
Bozdogan's Criterion (CAIC)	2384,883
Schwarz's Bayesian Criterion (BIC)	2382,883

The information criteria are displayed in smaller-is-better form.

a. Dependent Variable: hvas.

Fixed Effects

Type III Tests of Fixed Effects[a]

Source	Numerator df	Denominator df	F	Sig.
Intercept	1	,000	18,162	,000
time	1	265,000	,046	,830
ICDcontrol	1	265,000	,258	,612
time * ICDcontrol	1	265,000	,035	,851

a. Dependent Variable: hvas.

Estimates of Fixed Effects[a]

						95% Confidence Interval	
Parameter	Estimate	Std. Error	df	t	Sig.	Lower Bound	Upper Bound
Intercept	61,098361	14,788405	,000	4,132	,000	29,962480	92,234241
[time=1]	1,015925	3,604563	265,000	,282	,778	-6,081303	8,113153
[time=2]	0[b]	0	.	.	.	.	.
[ICDcontrol=1]	1,754098	3,726328	265,000	,471	,638	-5,582878	9,091075
[ICDcontrol=2]	0[b]	0	.	.	.	.	.
[time=1] * [ICDcontrol=1]	-,946306	5,043386	265,000	-,188	,851	-10,876513	8,983901
[time=1] * [ICDcontrol=2]	0[b]	0	.	.	.	.	.
[time=2] * [ICDcontrol=1]	0[b]	0	.	.	.	.	.
[time=2] * [ICDcontrol=2]	0[b]	0	.	.	.	.	.

a. Dependent Variable: hvas.

b. This parameter is set to zero because it is redundant.

Covariance Parameters

Estimates of Covariance Parameters[a]

Parameter		Estimate	Std. Error
Residual		423,508302	36,792050
Intercept	Variance	211,754151	24296003999,808400

a. Dependent Variable: hvas.

Random Effect Covariance Structure (G)[a]

	Intercept
Intercept	211,754151

Variance Components

a. Dependent Variable: hvas.

Estimated Marginal Means

time * ICDcontrol[a]

					95% Confidence Interval	
time	ICDcontrol	Mean	Std. Error	df	Lower Bound	Upper Bound
1	Control	62,922	14,740	,000	23,082	102,762
	ICD	62,114	14,758	,000	25,905	98,324
2	Control	62,852	14,788	,000	31,717	93,988
	ICD	61,098	14,788	,000	29,962	92,234

a. Dependent Variable: hvas.

```
TEMPORARY.
SELECT IF (ICDcontrol<=2 AND exclusie.x="Nee").
MIXED GDS BY time ICDcontrol
  /CRITERIA=CIN(95) MXITER(100) MXSTEP(10) SCORING(1) SINGULAR(0.000000000001) HCONVERGE(0,
    ABSOLUTE) LCONVERGE(0, ABSOLUTE) PCONVERGE(0.000001, ABSOLUTE)
  /FIXED=time ICDcontrol time*ICDcontrol | SSTYPE(3)
  /METHOD=REML
  /PRINT=DESCRIPTIVES G  SOLUTION
  /RANDOM=INTERCEPT | COVTYPE(VC)
  /EMMEANS=TABLES(time*ICDcontrol) .
```

Mixed Model Analysis

Notes

Output Created		27-AUG-2020 14:59:41
Comments		
Input	Data	M:\HP\Consult\HARTZ\Timal\ICD2\ICD2_QOL_both_long.sav
	Active Dataset	DataSet1
	Filter	<none>
	Weight	<none>
	Split File	<none>
	N of Rows in Working Data File	342
Missing Value Handling	Definition of Missing	User-defined missing values are treated as missing.
	Cases Used	Statistics are based on all cases with valid data for all variables in the model.

Syntax		MIXED GDS BY time ICDcontrol<br>/CRITERIA=CIN(95)<br>MXITER(100) MXSTEP(10)<br>SCORING(1)<br>SINGULAR(0.000000000001)<br>HCONVERGE(0,<br>ABSOLUTE) LCONVERGE(0,<br>ABSOLUTE)<br>PCONVERGE(0.000001,<br>ABSOLUTE)<br>/FIXED=time ICDcontrol<br>time*ICDcontrol | SSTYPE(3)<br>/METHOD=REML<br>/PRINT=DESCRIPTIVES G<br>SOLUTION<br>/RANDOM=INTERCEPT | COVTYPE(VC)<br><br>/EMMEANS=TABLES(time*ICDco<br>ntrol) .
Resources	Processor Time	00:00:00,03
	Elapsed Time	00:00:00,03

[DataSet1] M:\HP\Consult\HARTZ\Timal\ICD2\ICD2_QOL_both_long.sav

Warnings

The final Hessian matrix is not positive definite although all convergence criteria are satisfied. The MIXED procedure continues despite this warning. Validity of subsequent results cannot be ascertained.

Descriptive Statistics

GDS

time	ICDcontrol	Count	Mean	Standard Deviation	Coefficient of Variation
1	Control	71	3,2394	3,05878	94,4%

	ICD	63	2,8571	2,76998	96,9%
	Total	134	3,0597	2,92194	95,5%
2	Control	61	3,0820	2,92287	94,8%
	ICD	61	3,0328	2,71396	89,5%
	Total	122	3,0574	2,80878	91,9%
Total	Control	132	3,1667	2,98640	94,3%
	ICD	124	2,9435	2,73282	92,8%
	Total	256	3,0586	2,86296	93,6%

Model Dimension[a]

		Number of Levels	Covariance Structure	Number of Parameters
Fixed Effects	Intercept	1		1
	time	2		1
	ICDcontrol	2		1
	time * ICDcontrol	4		1
Random Effects	Intercept[b]	1	Variance Components	1
Residual				1
Total		10		6

a. Dependent Variable: GDS.

b. As of version 11.5, the syntax rules for the RANDOM subcommand have changed. Your command syntax may yield results that differ from those produced by prior versions. If you are using version 11 syntax, please consult the current syntax reference guide for more information.

Information Criteria[a]

-2 Restricted Log Likelihood	1264,293
Akaike's Information Criterion (AIC)	1268,293
Hurvich and Tsai's Criterion (AICC)	1268,341
Bozdogan's Criterion (CAIC)	1277,352
Schwarz's Bayesian Criterion (BIC)	1275,352

The information criteria are displayed in smaller-is-better form.

a. Dependent Variable: GDS.

Fixed Effects

Type III Tests of Fixed Effects[a]

Source	Numerator df	Denominator df	F	Sig.
Intercept	1	252	287,217	,000
time	1	252	,001	,980
ICDcontrol	1	252	,359	,550
time * ICDcontrol	1	252	,214	,644

a. Dependent Variable: GDS.

Estimates of Fixed Effects[a]

						95% Confidence Interval	
Parameter	Estimate	Std. Error	df	t	Sig.	Lower Bound	Upper Bound
Intercept	3,032787	,368303	252	8,234	,000	2,307443	3,758131
[time=1]	-,175644	,516709	252	-,340	,734	-1,193262	,841974
[time=2]	0[b]	0	.	.	.	.	.
[ICDcontrol=1]	,049180	,520859	252	,094	,925	-,976612	1,074972
[ICDcontrol=2]	0[b]	0	.	.	.	.	.
[time=1] * [ICDcontrol=1]	,333113	,720540	252	,462	,644	-1,085933	1,752160
[time=1] * [ICDcontrol=2]	0[b]	0	.	.	.	.	.
[time=2] * [ICDcontrol=1]	0[b]	0	.	.	.	.	.
[time=2] * [ICDcontrol=2]	0[b]	0	.	.	.	.	.

a. Dependent Variable: GDS.

b. This parameter is set to zero because it is redundant.

Covariance Parameters

Estimates of Covariance Parameters[a]

Parameter		Estimate	Std. Error
Residual		8,274478	,737149
Intercept	Variance	,000000[b]	,000000

a. Dependent Variable: GDS.

b. This covariance parameter is redundant.

Random Effect Covariance Structure (G)[a]

	Intercept
Intercept	,000000

Variance Components

a. Dependent Variable: GDS.

Estimated Marginal Means

time * ICDcontrol[a]

					95% Confidence Interval	
time	ICDcontrol	Mean	Std. Error	df	Lower Bound	Upper Bound
1	Control	3,239	,341	252	2,567	3,912
	ICD	2,857	,362	252	2,143	3,571
2	Control	3,082	,368	252	2,357	3,807
	ICD	3,033	,368	252	2,307	3,758

a. Dependent Variable: GDS.

```
TEMPORARY.
SELECT IF (ICDcontrol<=2 AND exclusie.x="Nee").
MIXED LOTR BY time ICDcontrol
  /CRITERIA=CIN(95) MXITER(100) MXSTEP(10) SCORING(1) SINGULAR(0.000000000001) HCONVERGE(0,
    ABSOLUTE) LCONVERGE(0, ABSOLUTE) PCONVERGE(0.000001, ABSOLUTE)
  /FIXED=time ICDcontrol time*ICDcontrol | SSTYPE(3)
  /METHOD=REML
  /PRINT=DESCRIPTIVES G  SOLUTION
```

```
/RANDOM=INTERCEPT | COVTYPE(VC)
/EMMEANS=TABLES(time*ICDcontrol) .
```

Mixed Model Analysis

Notes

Output Created		27-AUG-2020 14:59:41
Comments		
Input	Data	M:\HP\Consult\HARTZ\Timal\ICD2\ICD2_QOL_both_long.sav
	Active Dataset	DataSet1
	Filter	<none>
	Weight	<none>
	Split File	<none>
	N of Rows in Working Data File	342
Missing Value Handling	Definition of Missing	User-defined missing values are treated as missing.
	Cases Used	Statistics are based on all cases with valid data for all variables in the model.

Syntax		MIXED LOTR BY time ICDcontrol<br>/CRITERIA=CIN(95) MXITER(100) MXSTEP(10) SCORING(1) SINGULAR(0.000000000001) HCONVERGE(0, ABSOLUTE) LCONVERGE(0, ABSOLUTE) PCONVERGE(0.000001, ABSOLUTE)<br>/FIXED=time ICDcontrol time*ICDcontrol | SSTYPE(3)<br>/METHOD=REML<br>/PRINT=DESCRIPTIVES G SOLUTION<br>/RANDOM=INTERCEPT | COVTYPE(VC)<br>/EMMEANS=TABLES(time*ICDcontrol) .
Resources	Processor Time	00:00:00,08
	Elapsed Time	00:00:00,04

Warnings

The final Hessian matrix is not positive definite although all convergence criteria are satisfied. The MIXED procedure continues despite this warning. Validity of subsequent results cannot be ascertained.

Descriptive Statistics

Totale score voor LOT-R berekenen at baseline

time	ICDcontrol	Count	Mean	Standard Deviation	Coefficient of Variation
1	Control	71	14,73	3,355	22,8%
	ICD	61	14,51	3,409	23,5%
	Total	132	14,63	3,369	23,0%
2	Control	60	13,58	3,099	22,8%

	ICD	61	14,59	3,019	20,7%
	Total	121	14,09	3,088	21,9%
Total	Control	131	14,21	3,279	23,1%
	ICD	122	14,55	3,207	22,0%
	Total	253	14,37	3,242	22,6%

Model Dimension[a]

		Number of Levels	Covariance Structure	Number of Parameters
Fixed Effects	Intercept	1		1
	time	2		1
	ICDcontrol	2		1
	time * ICDcontrol	4		1
Random Effects	Intercept[b]	1	Variance Components	1
Residual				1
Total		10		6

a. Dependent Variable: Totale score voor LOT-R berekenen at baseline.

b. As of version 11.5, the syntax rules for the RANDOM subcommand have changed. Your command syntax may yield results that differ from those produced by prior versions. If you are using version 11 syntax, please consult the current syntax reference guide for more information.

Information Criteria[a]

-2 Restricted Log Likelihood	1307,174
Akaike's Information Criterion (AIC)	1311,174
Hurvich and Tsai's Criterion (AICC)	1311,223
Bozdogan's Criterion (CAIC)	1320,209
Schwarz's Bayesian Criterion (BIC)	1318,209

The information criteria are displayed in smaller-is-better form.

a. Dependent Variable: Totale score voor LOT-R berekenen at baseline.

Fixed Effects

Type III Tests of Fixed Effects[a]

Source	Numerator df	Denominator df	F	Sig.
Intercept	1	249	4971,413	,000
time	1	249	1,717	,191
ICDcontrol	1	249	,924	,337
time * ICDcontrol	1	249	2,285	,132

a. Dependent Variable: Totale score voor LOT-R berekenen at baseline.

Estimates of Fixed Effects[a]

Parameter	Estimate	Std. Error	df	t	Sig.	95% Confidence Interval	
						Lower Bound	Upper Bound
Intercept	14,590164	,413615	249	35,275	,000	13,775533	15,404795
[time=1]	-,081967	,584940	249	-,140	,889	-1,234029	1,070095
[time=2]	0[b]	0	.	.	.	.	.
[ICDcontrol=1]	-1,006831	,587373	249	-1,714	,088	-2,163683	,150022
[ICDcontrol=2]	0[b]	0	.	.	.	.	.
[time=1] * [ICDcontrol=1]	1,231028	,814289	249	1,512	,132	-,372744	2,834800
[time=1] * [ICDcontrol=2]	0[b]	0	.	.	.	.	.
[time=2] * [ICDcontrol=1]	0[b]	0	.	.	.	.	.
[time=2] * [ICDcontrol=2]	0[b]	0	.	.	.	.	.

a. Dependent Variable: Totale score voor LOT-R berekenen at baseline.

b. This parameter is set to zero because it is redundant.

Covariance Parameters

Estimates of Covariance Parameters[a]

Parameter	Estimate	Std. Error
Residual	10,435738	,935273

Intercept	Variance	1,609893E-17[b]	,000000

a. Dependent Variable: Totale score voor LOT-R berekenen at baseline.

b. This covariance parameter is redundant.

Random Effect Covariance Structure (G)[a]

	Intercept
Intercept	1,609893E-17

Variance Components

a. Dependent Variable: Totale score voor LOT-R berekenen at baseline.

Estimated Marginal Means

time * ICDcontrol[a]

					95% Confidence Interval	
time	ICDcontrol	Mean	Std. Error	df	Lower Bound	Upper Bound
1	Control	14,732	,383	249	13,977	15,487
	ICD	14,508	,414	249	13,694	15,323
2	Control	13,583	,417	249	12,762	14,405
	ICD	14,590	,414	249	13,776	15,405

a. Dependent Variable: Totale score voor LOT-R berekenen at baseline.
